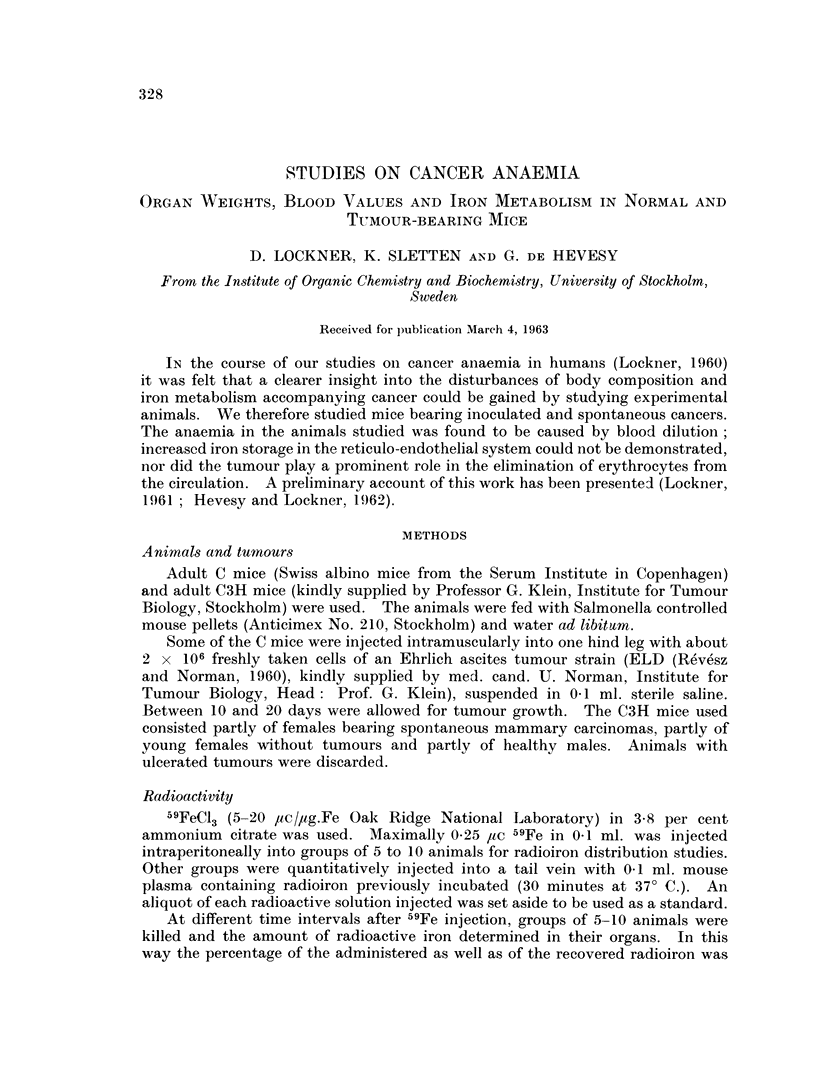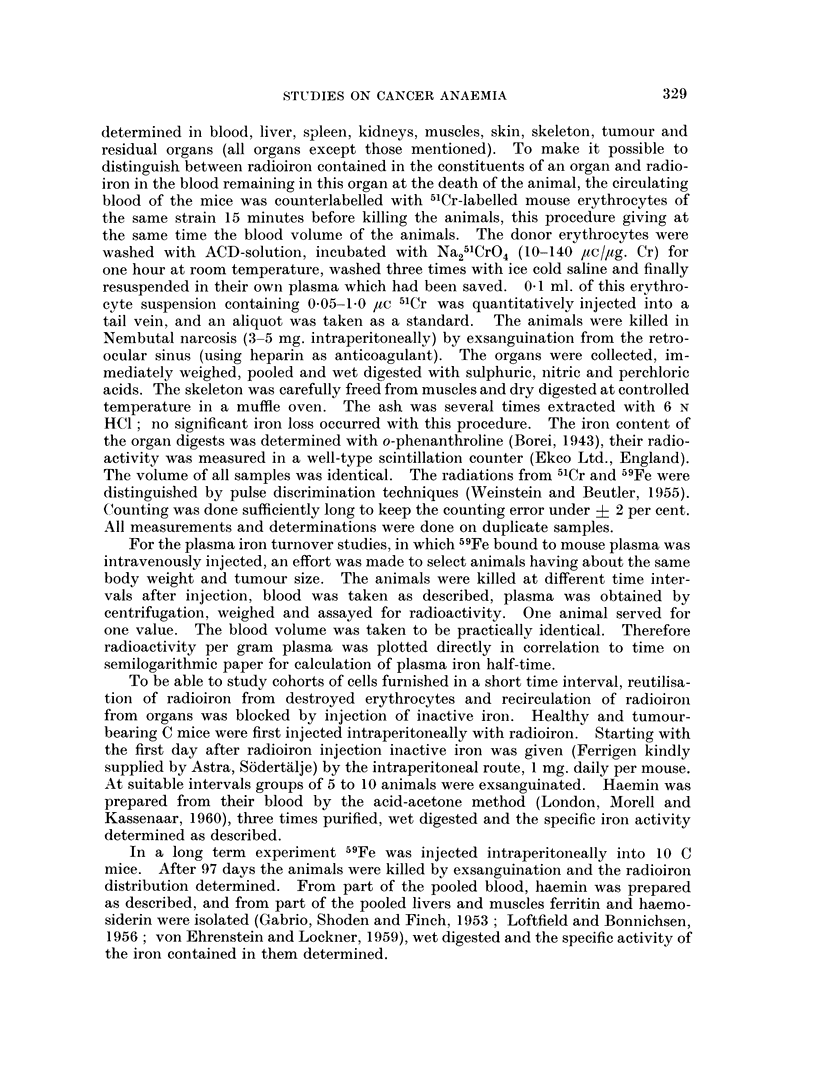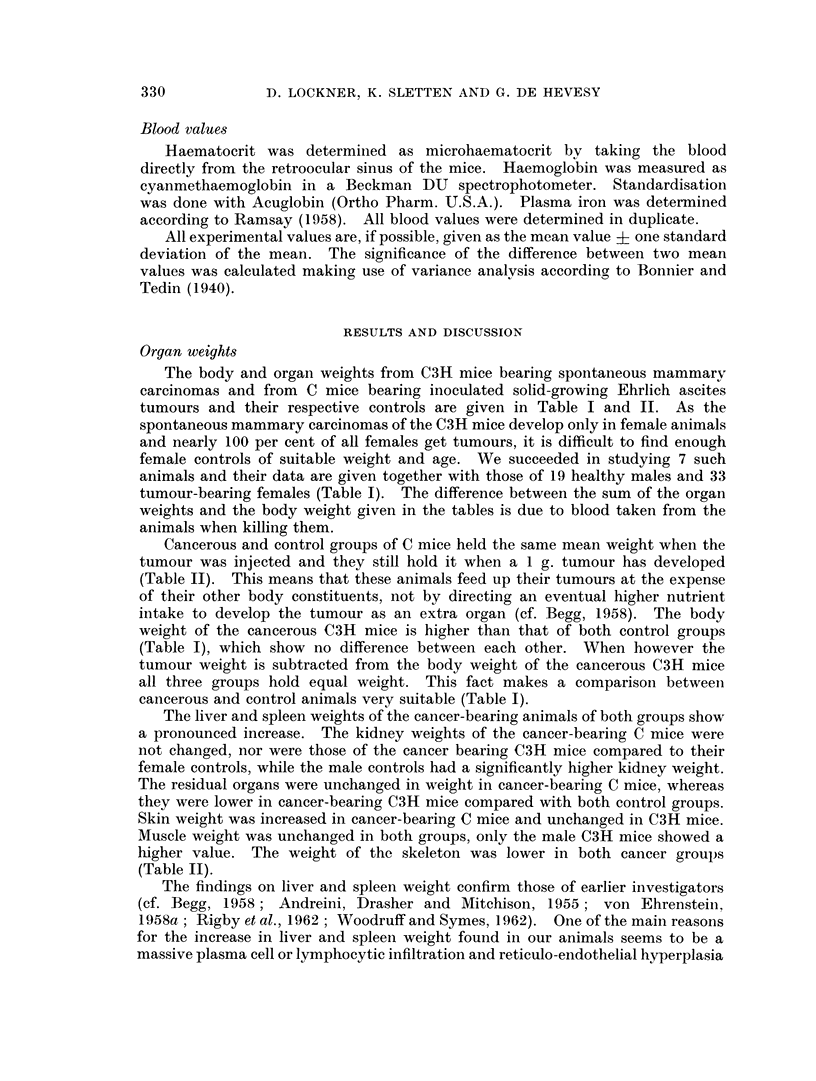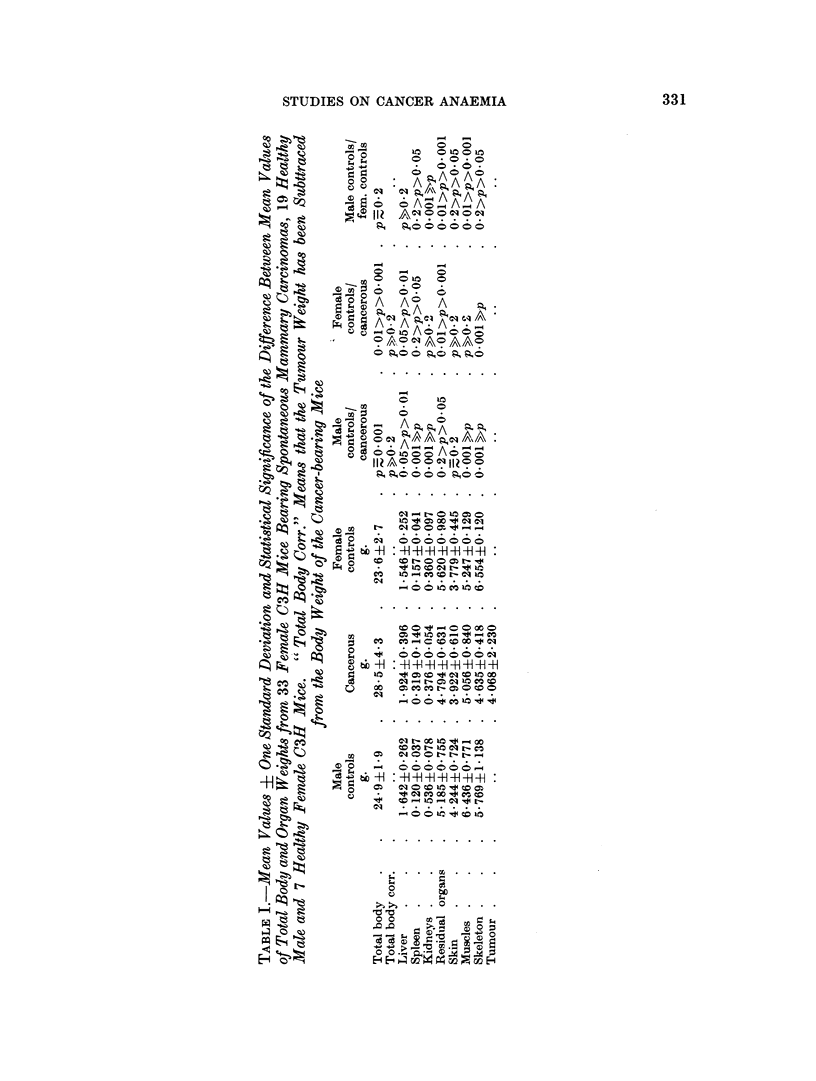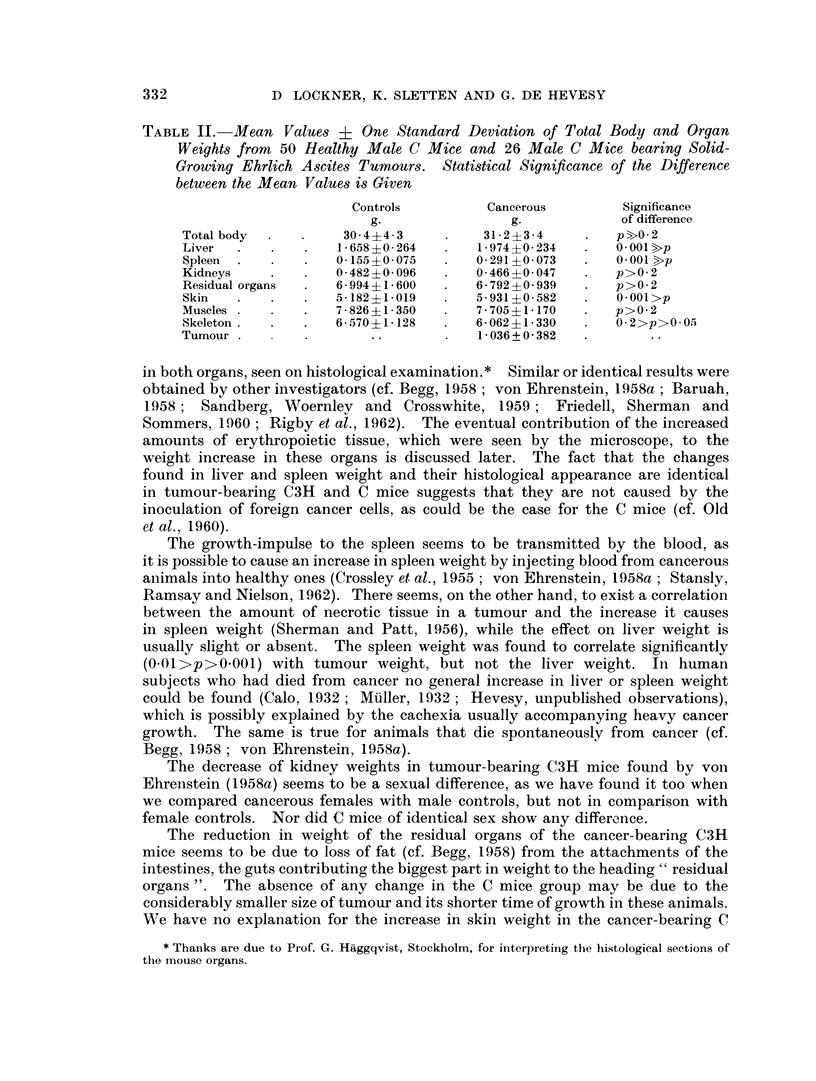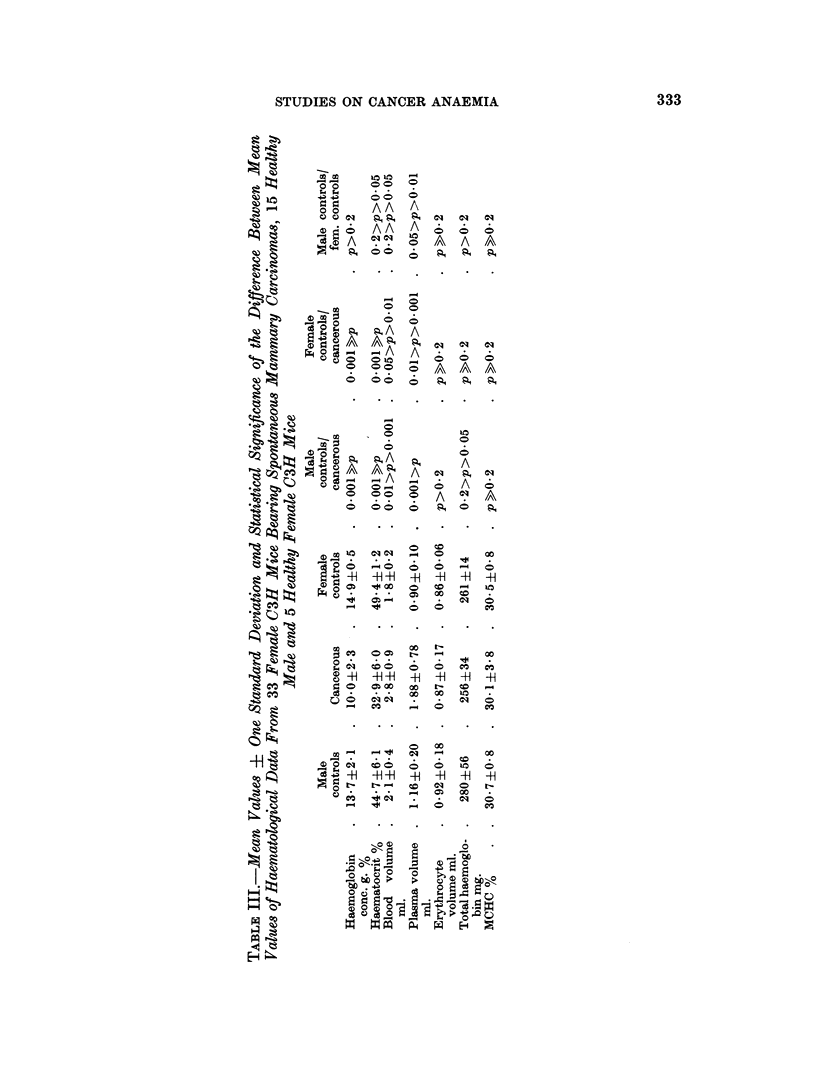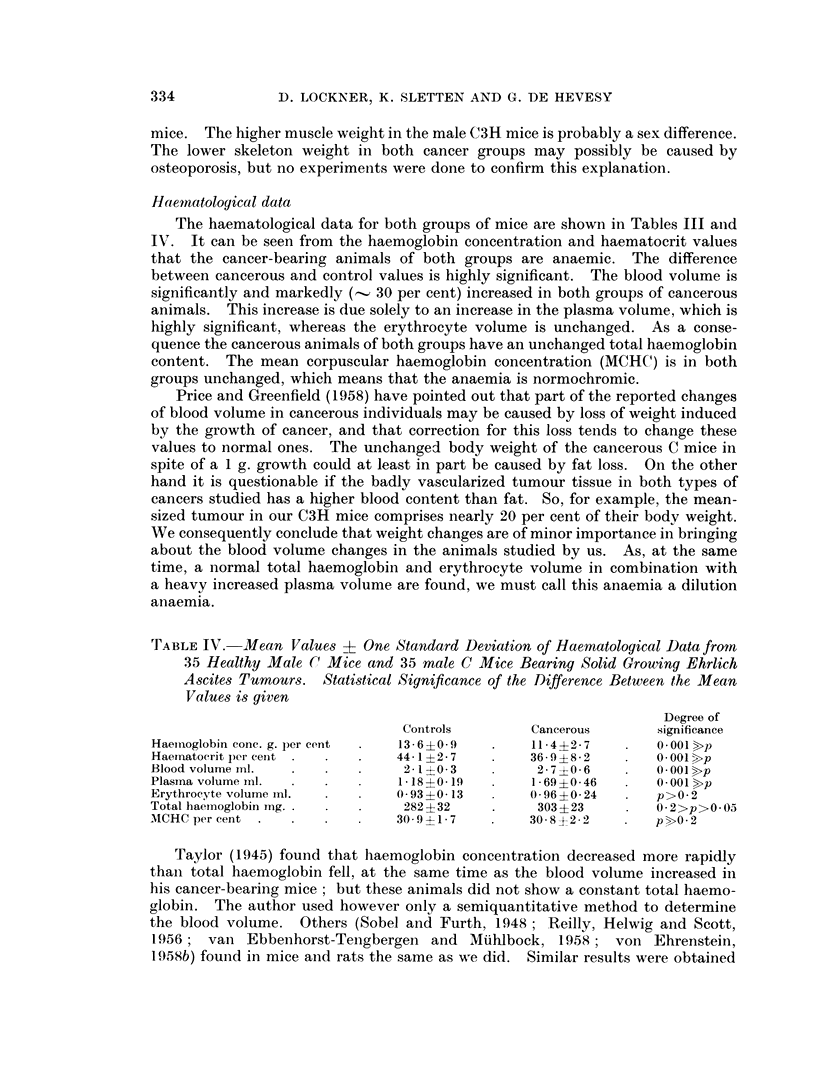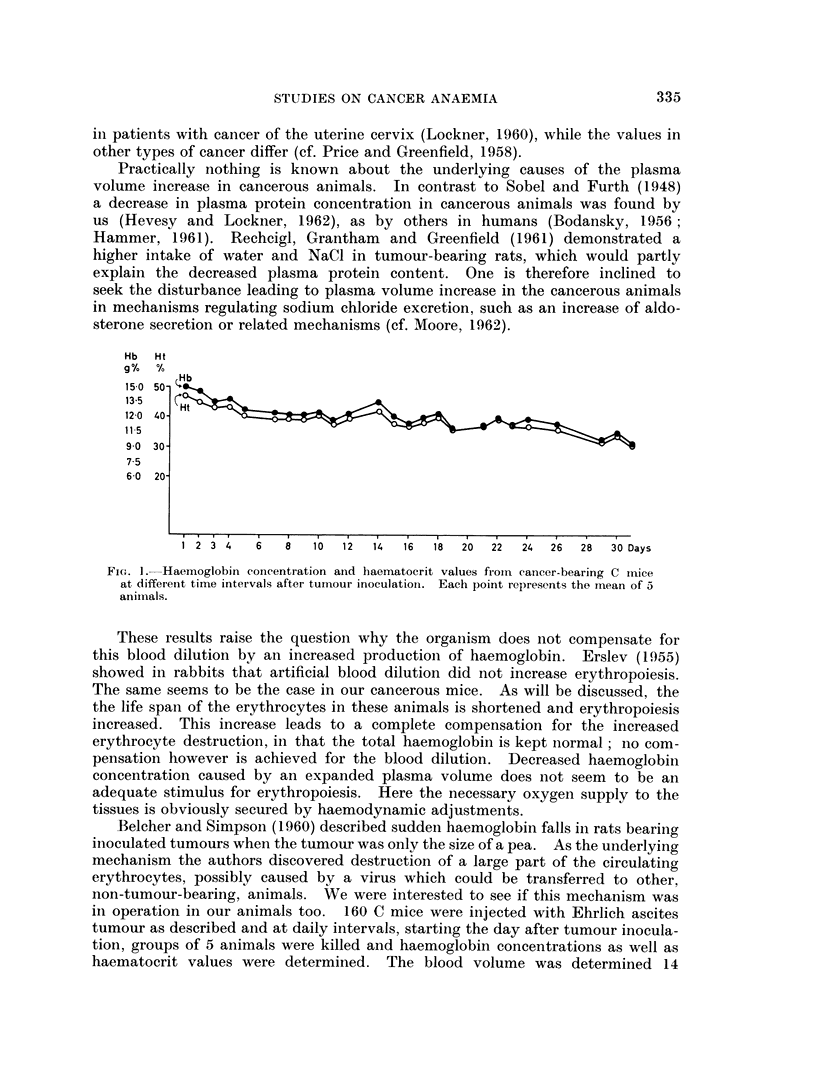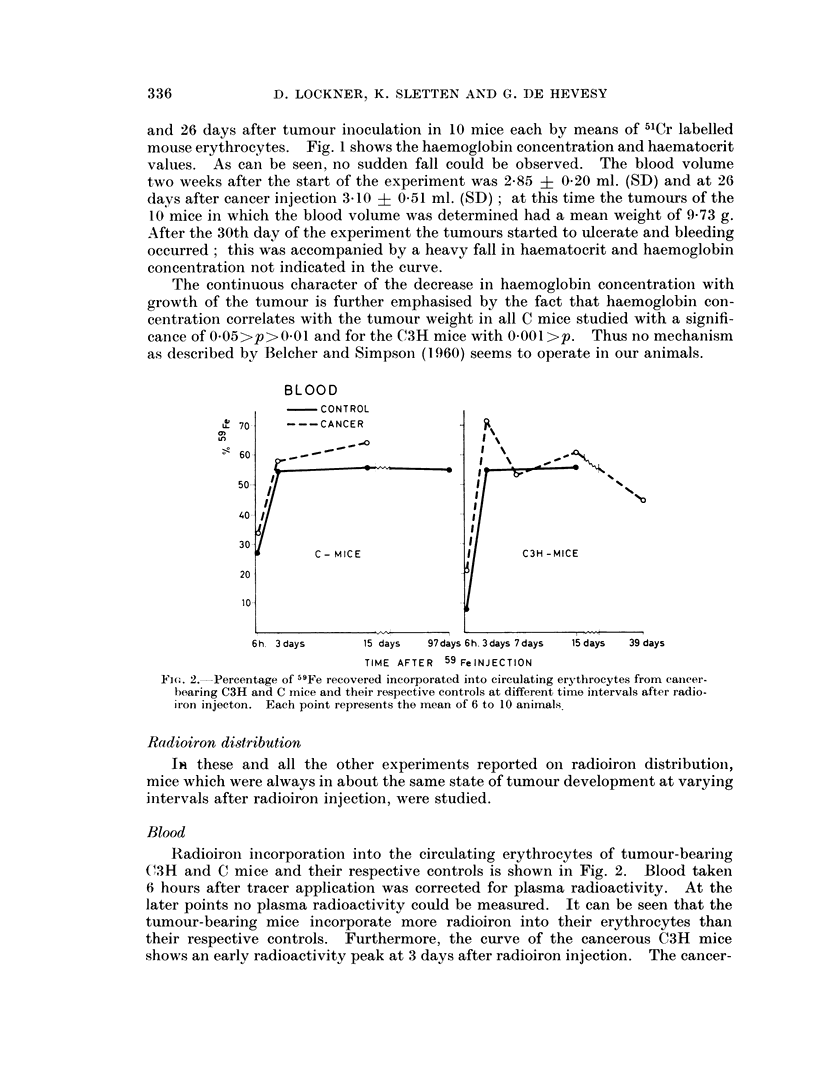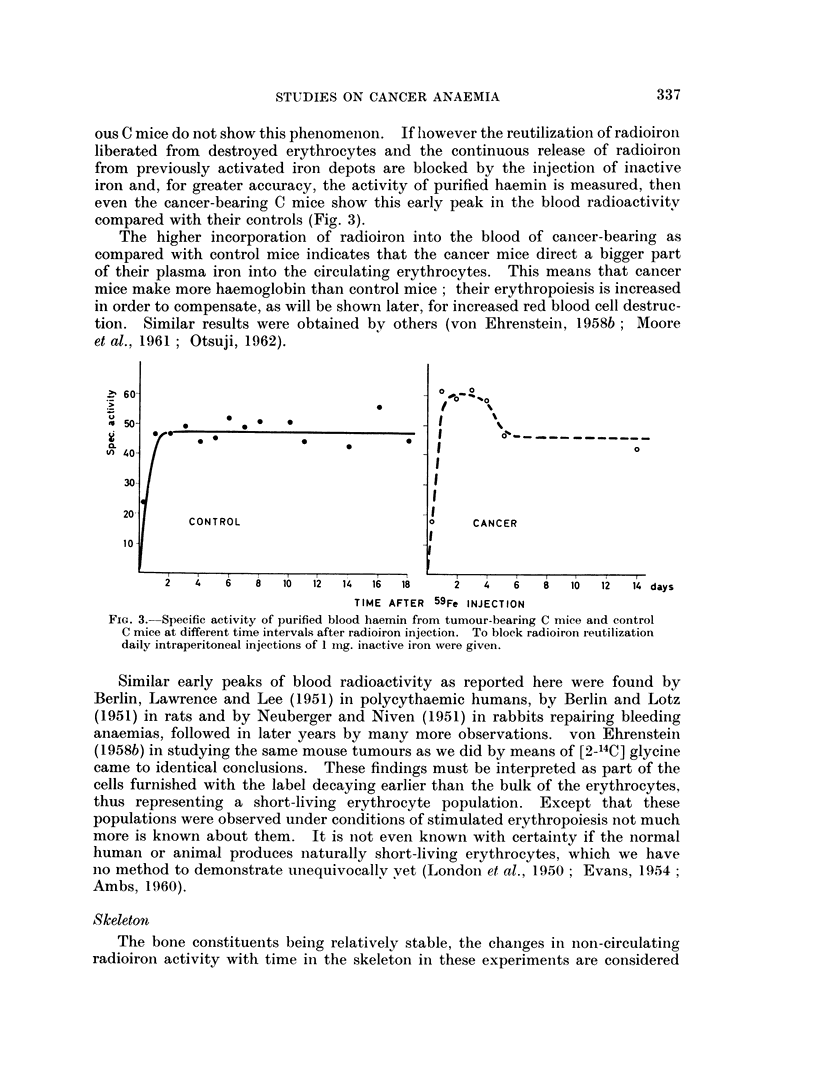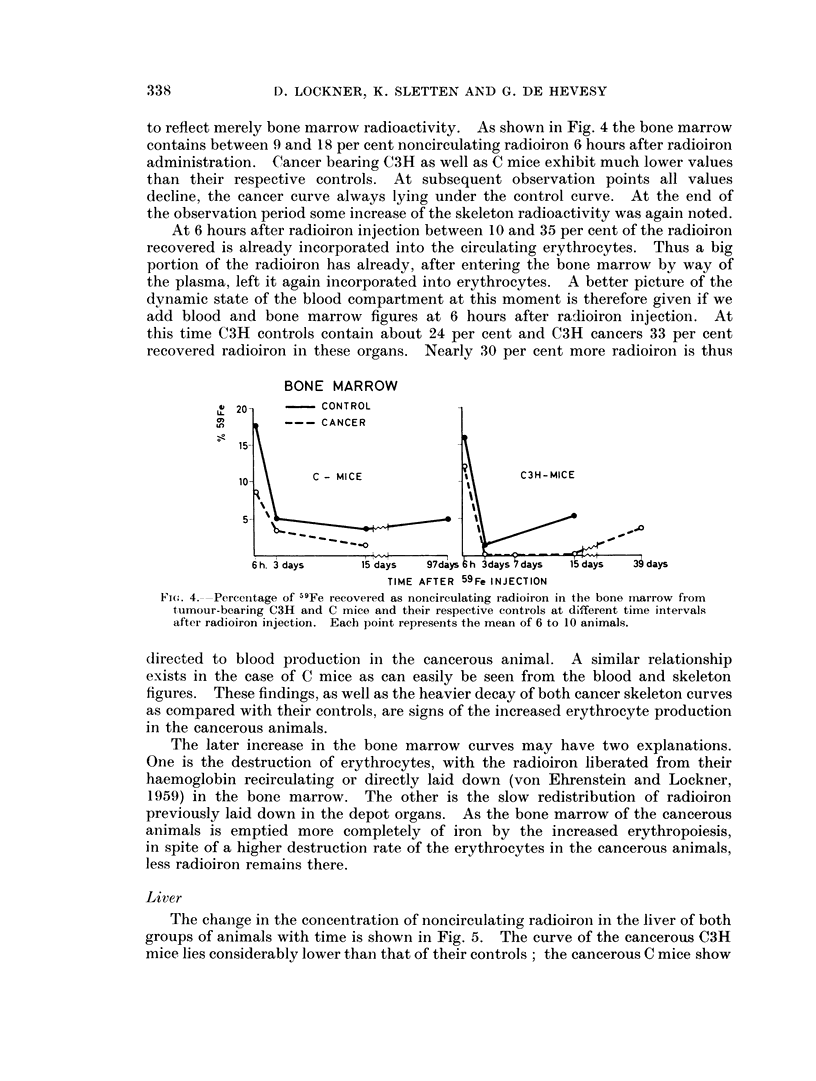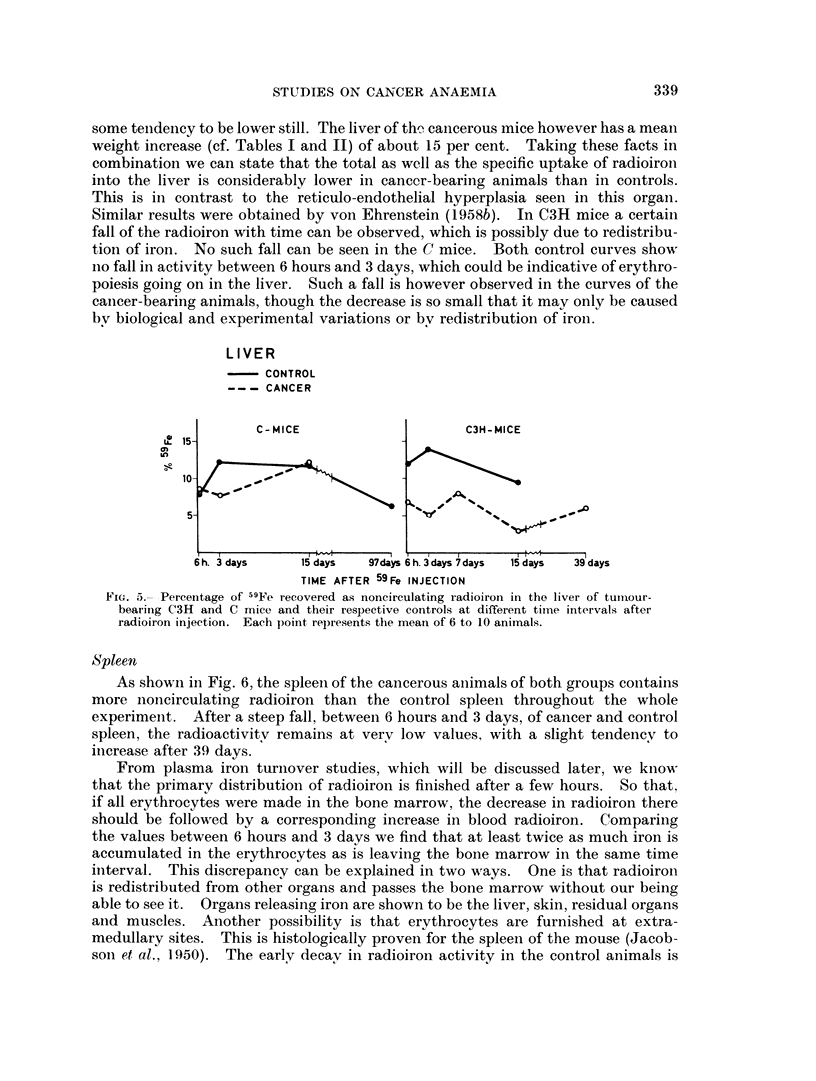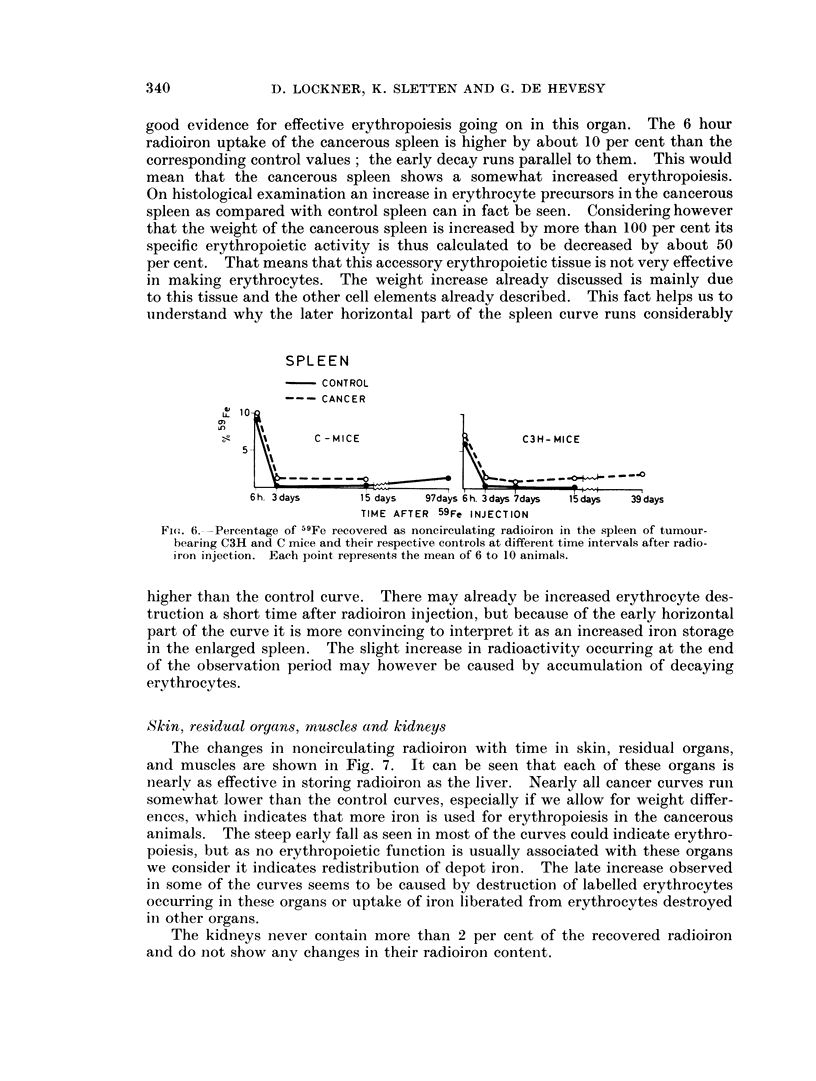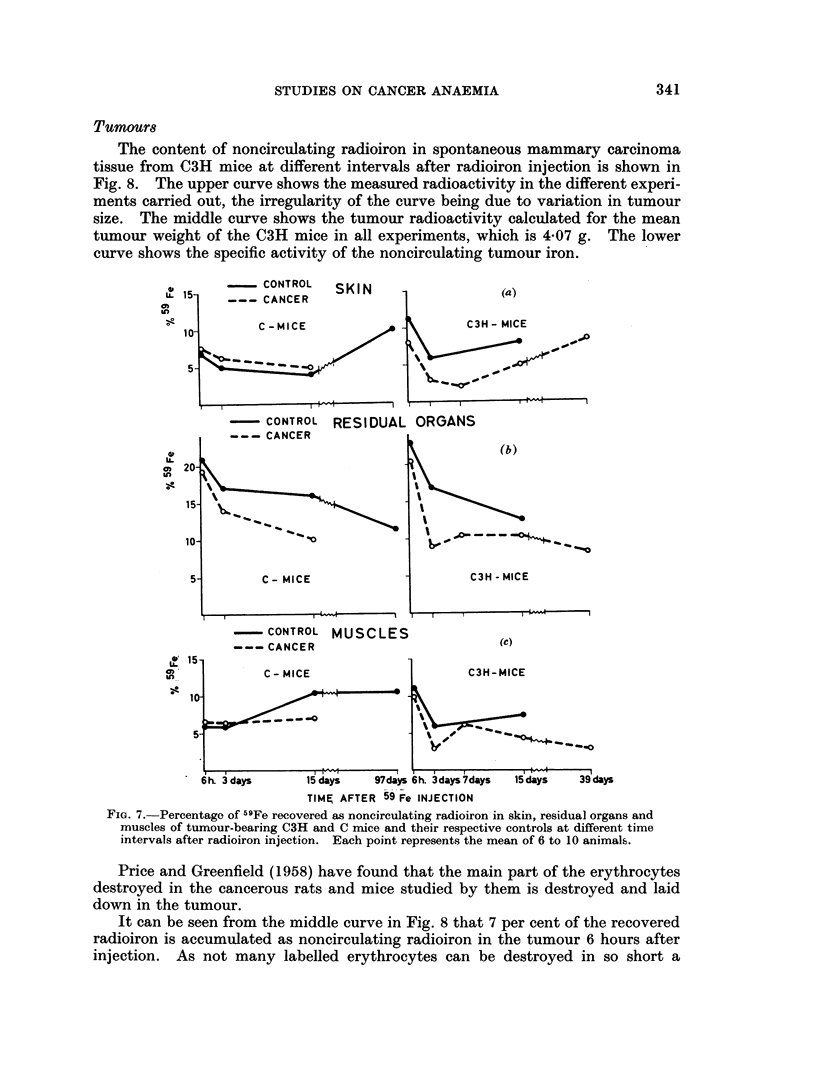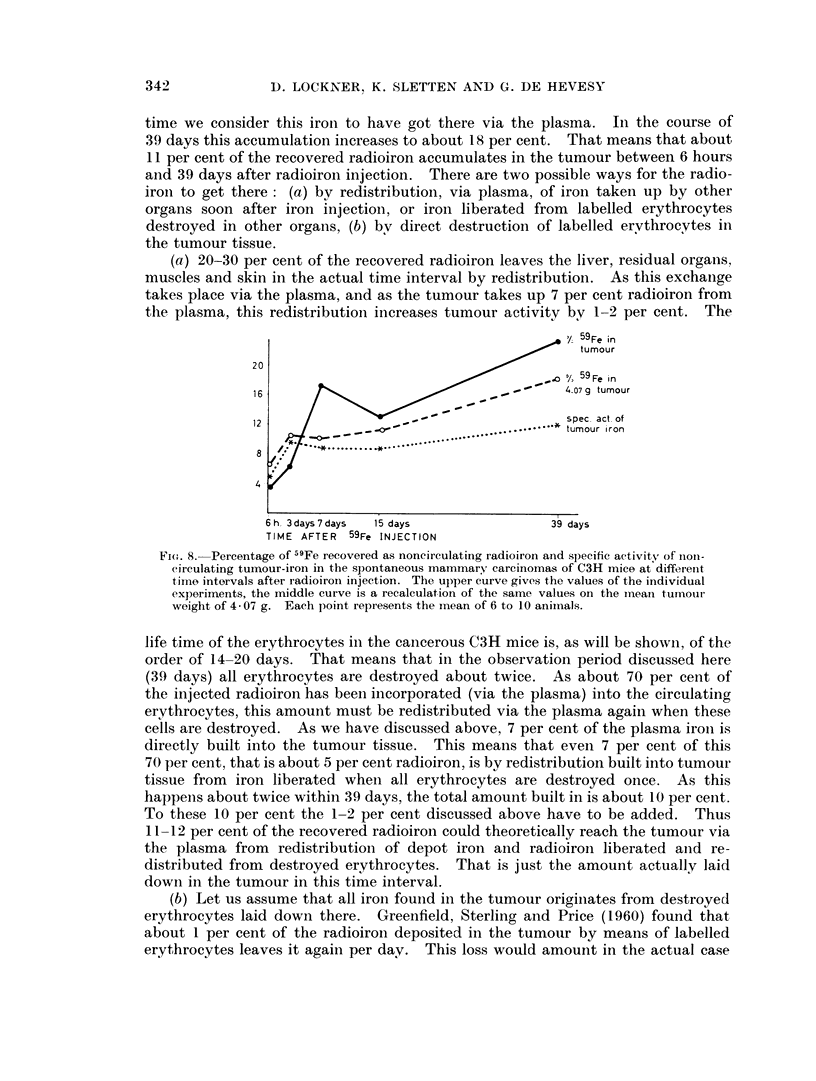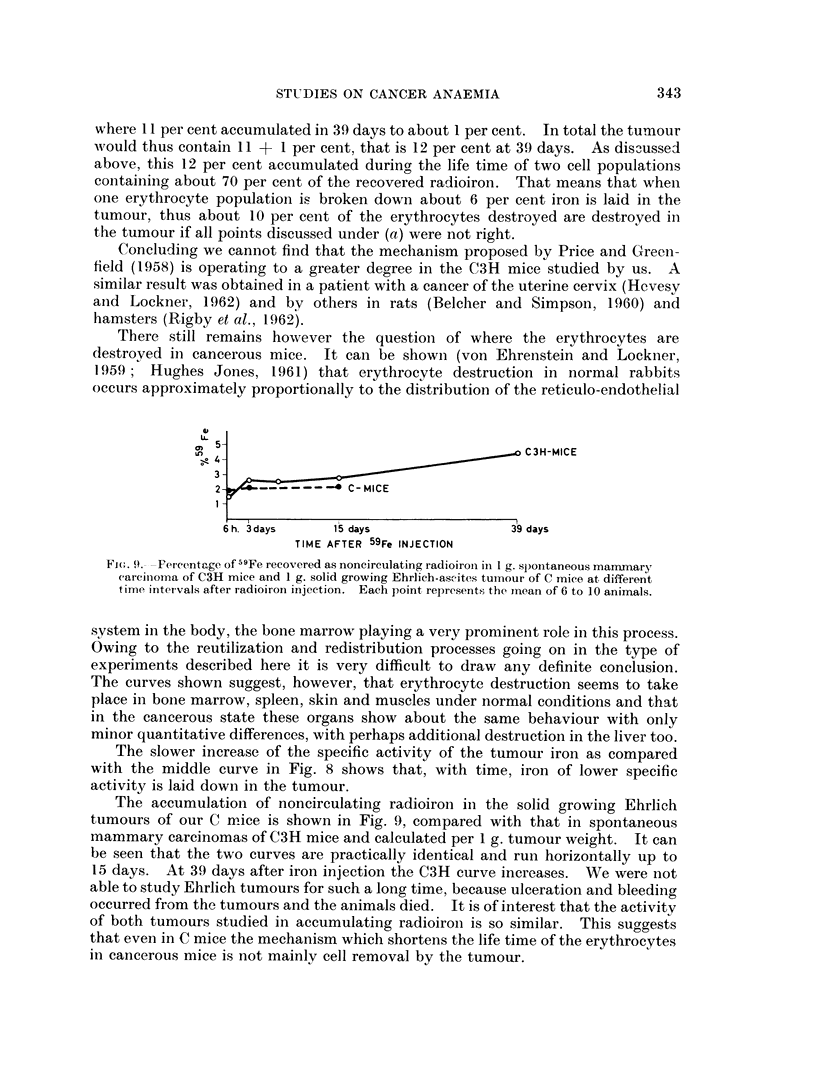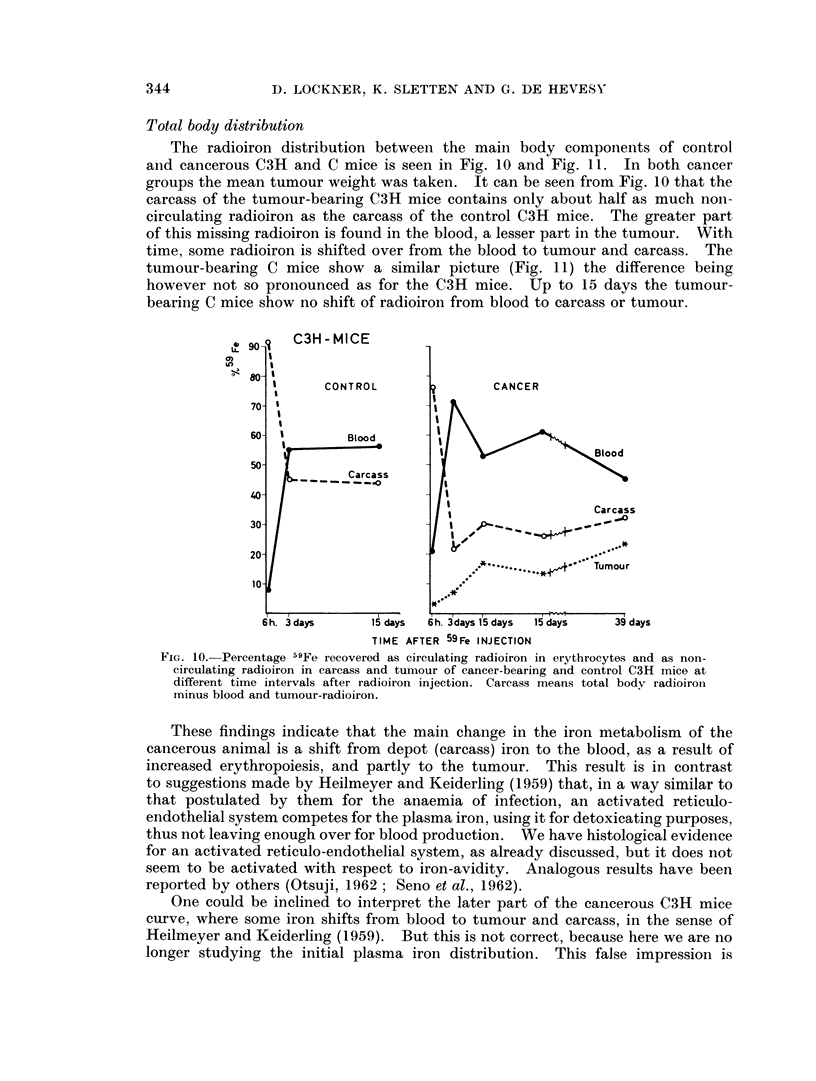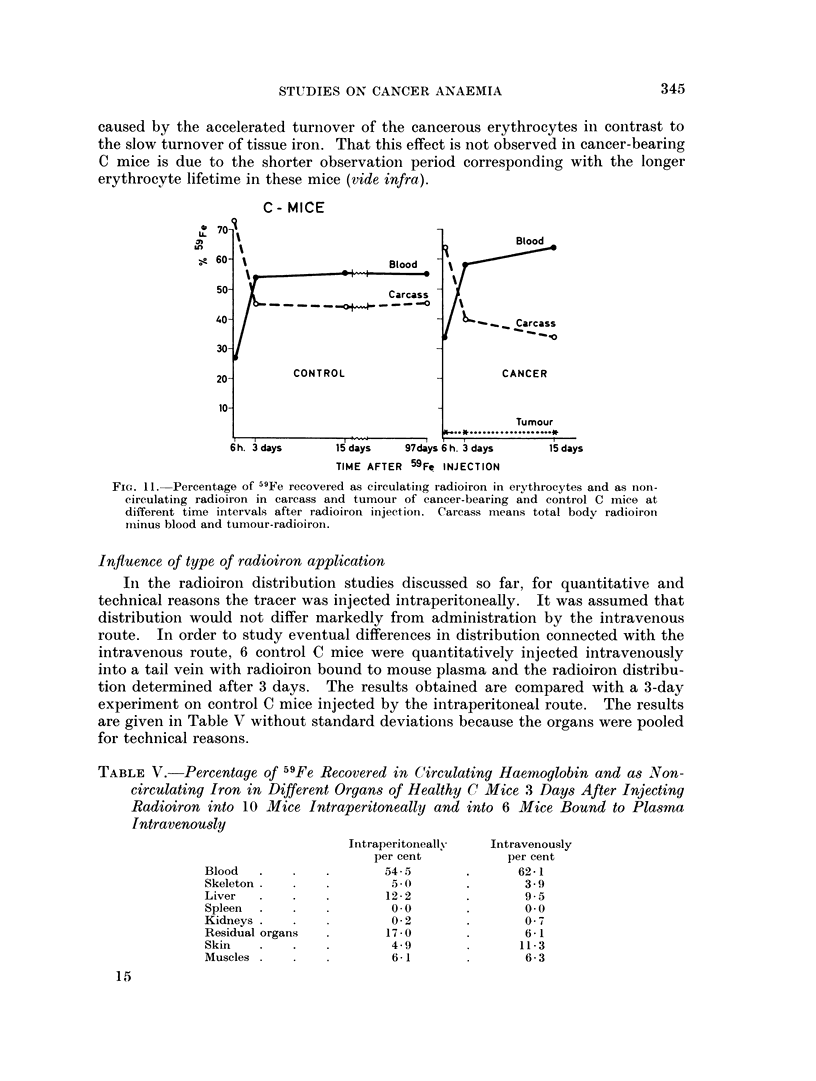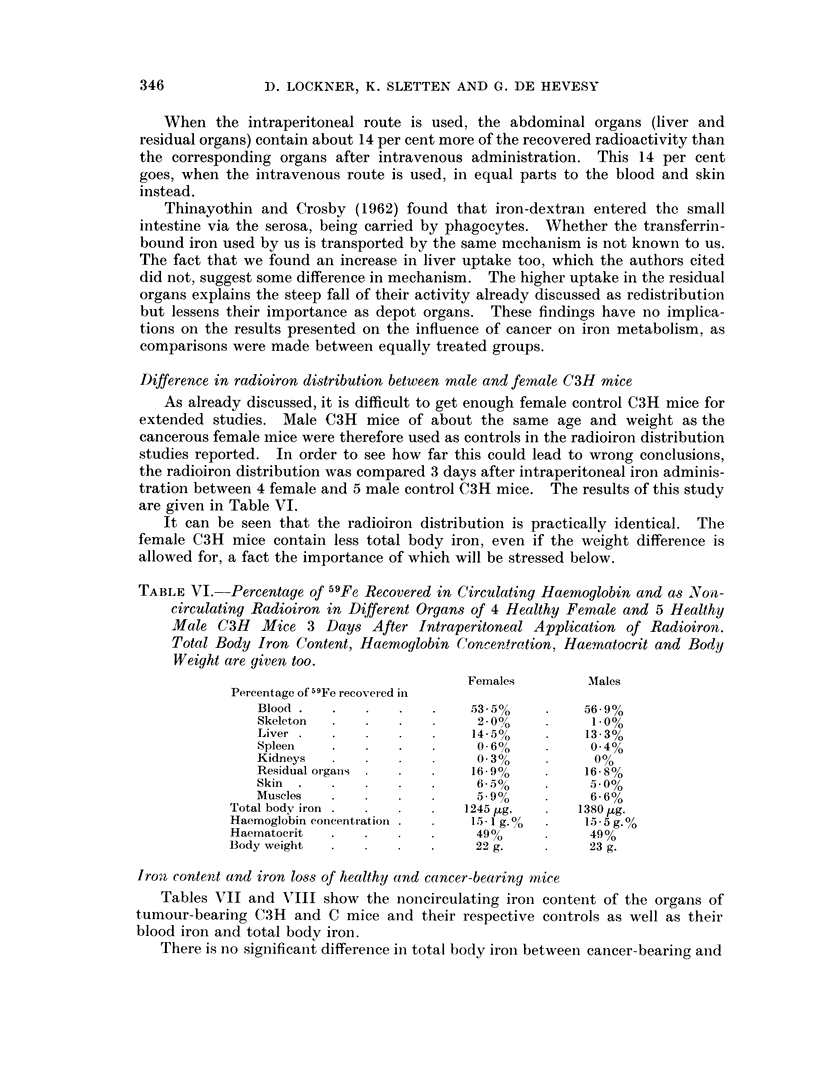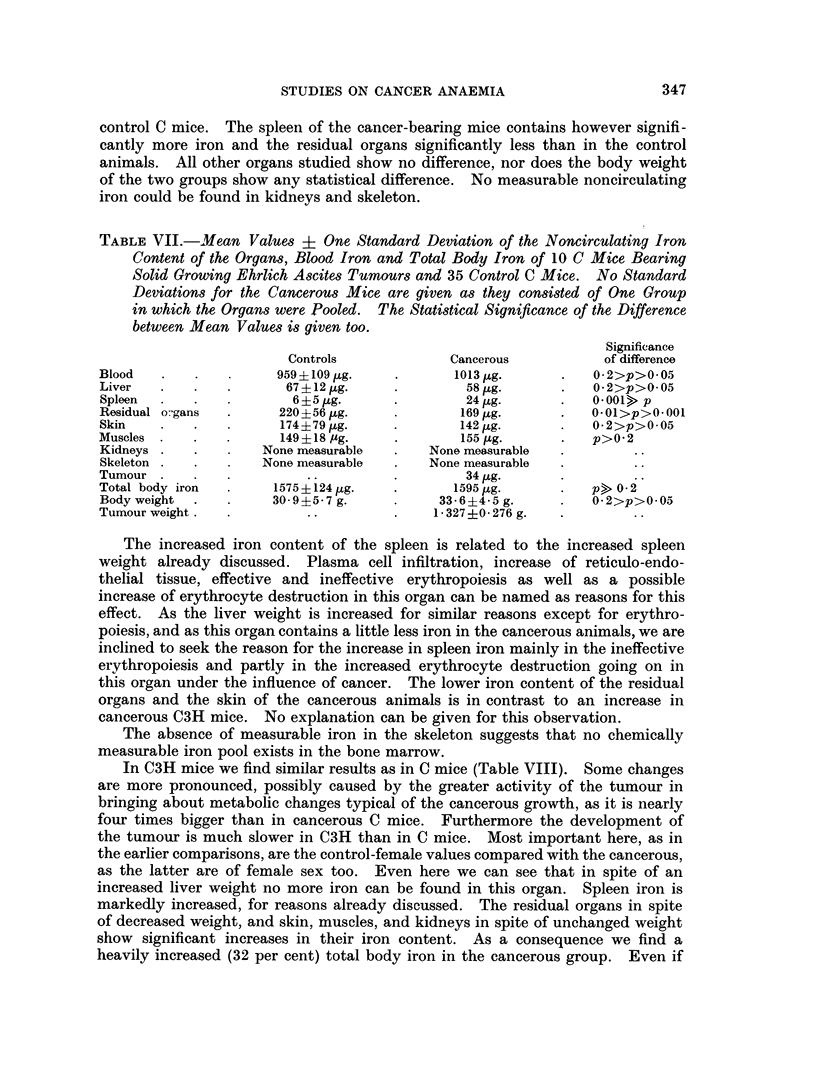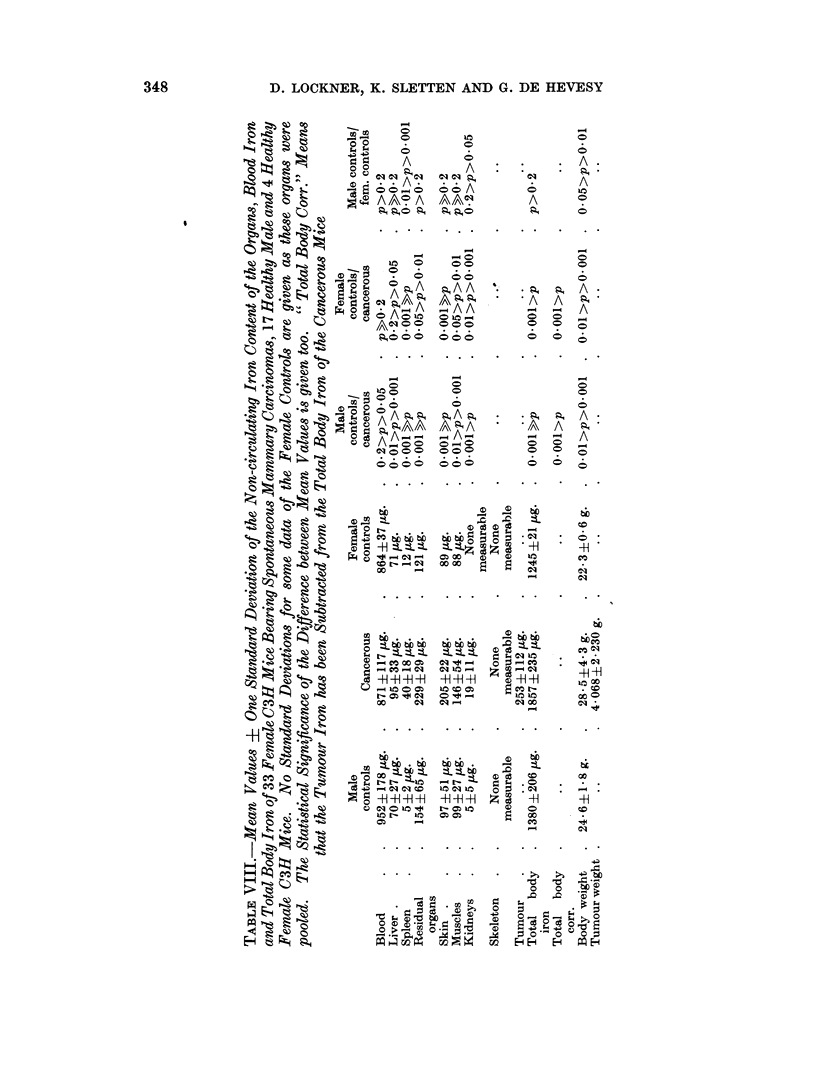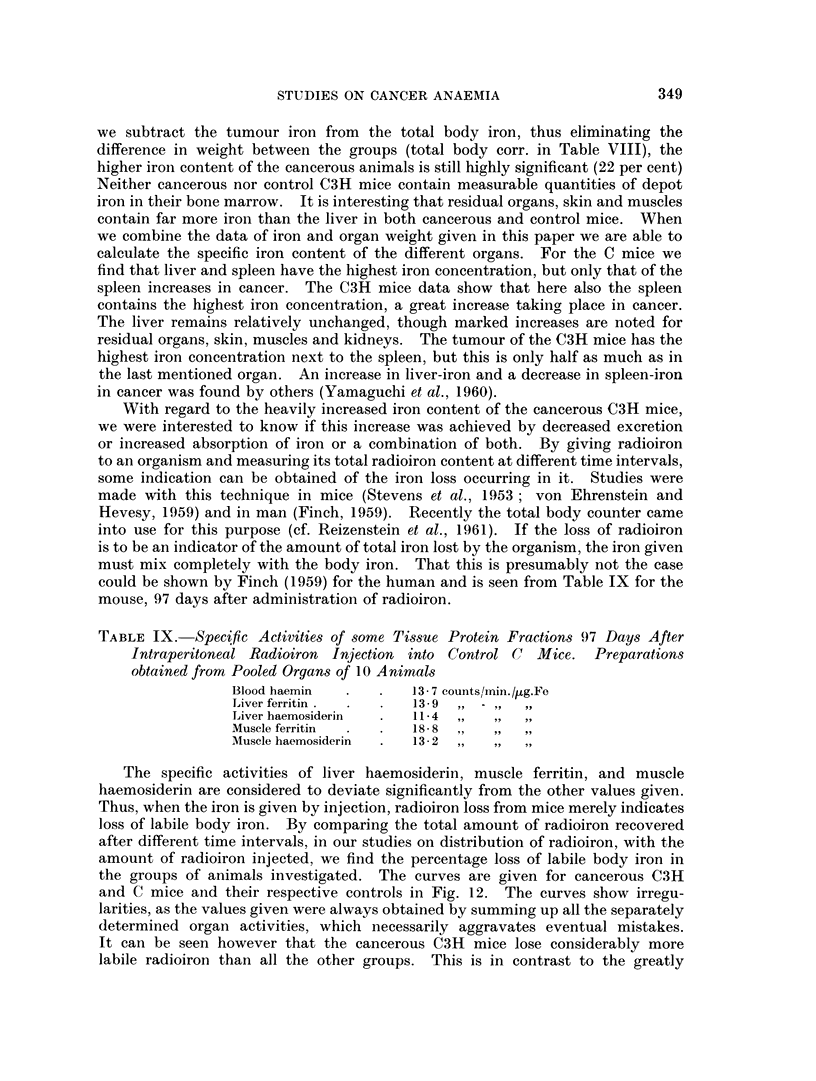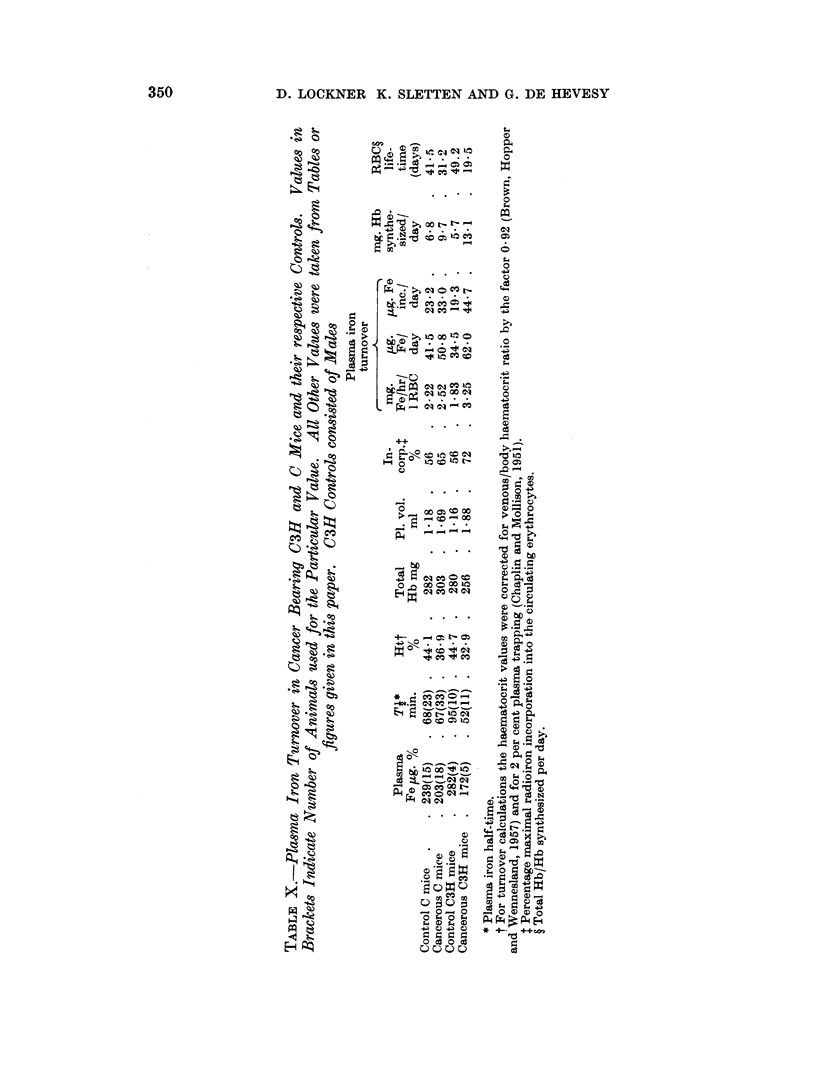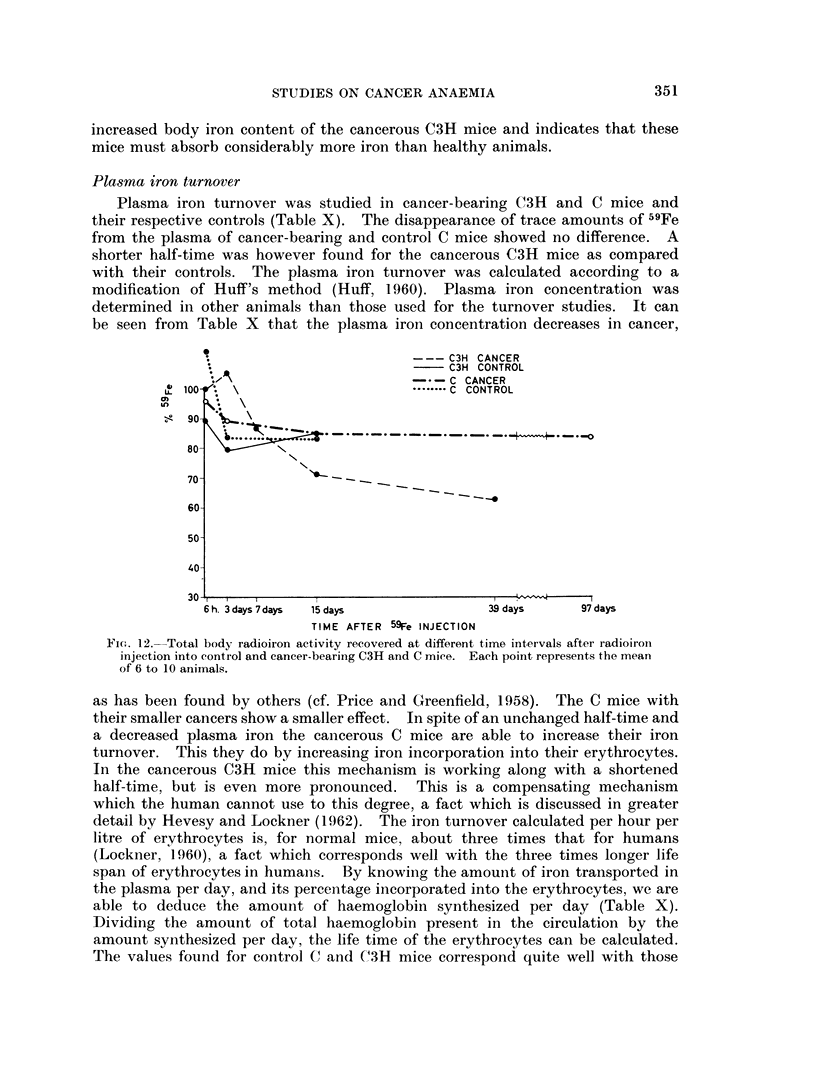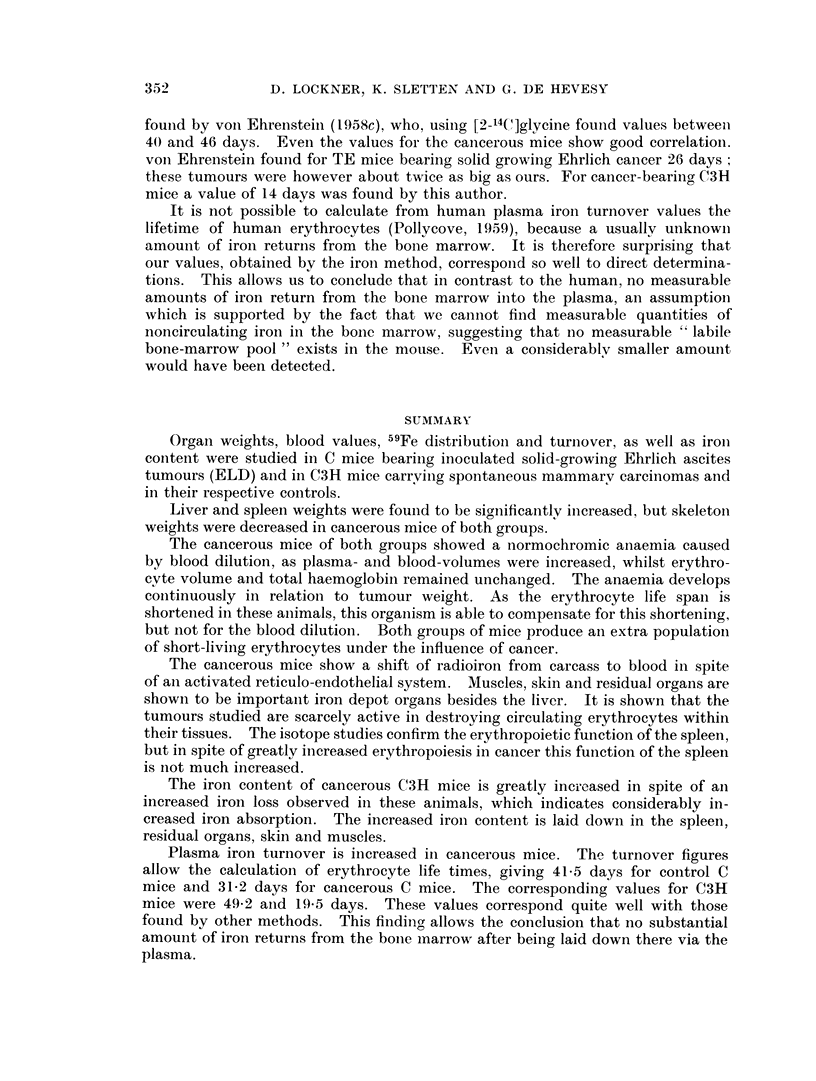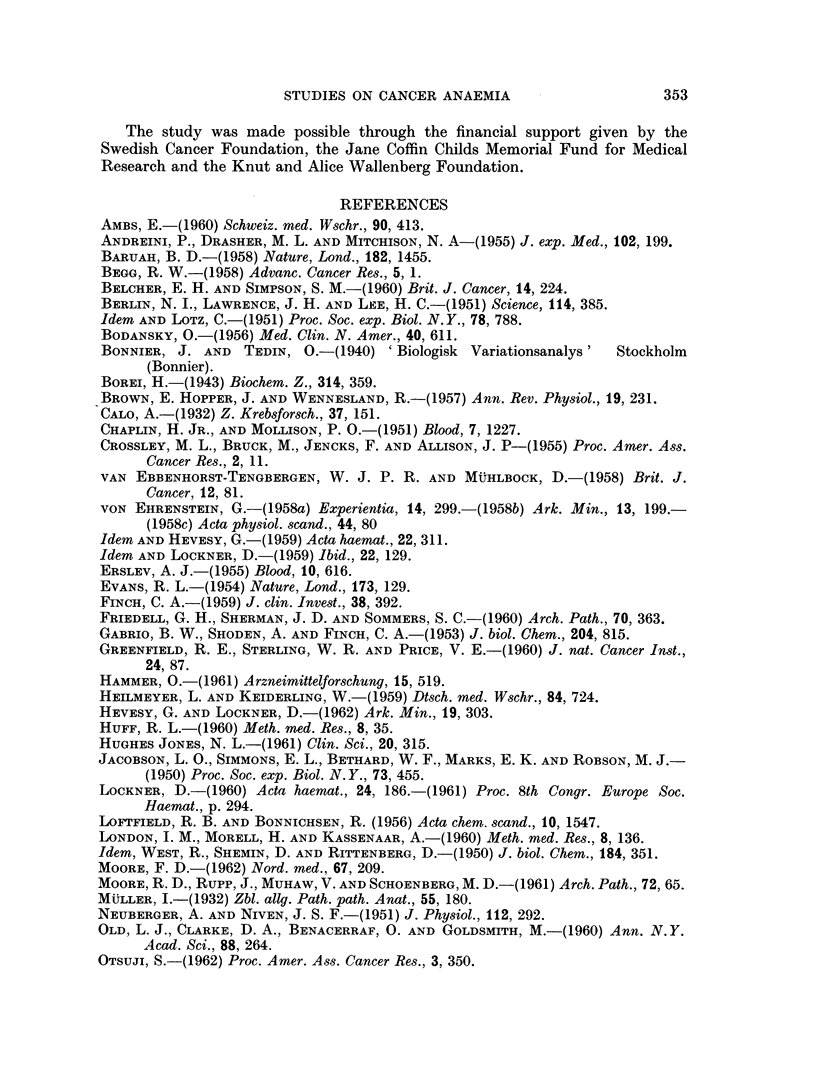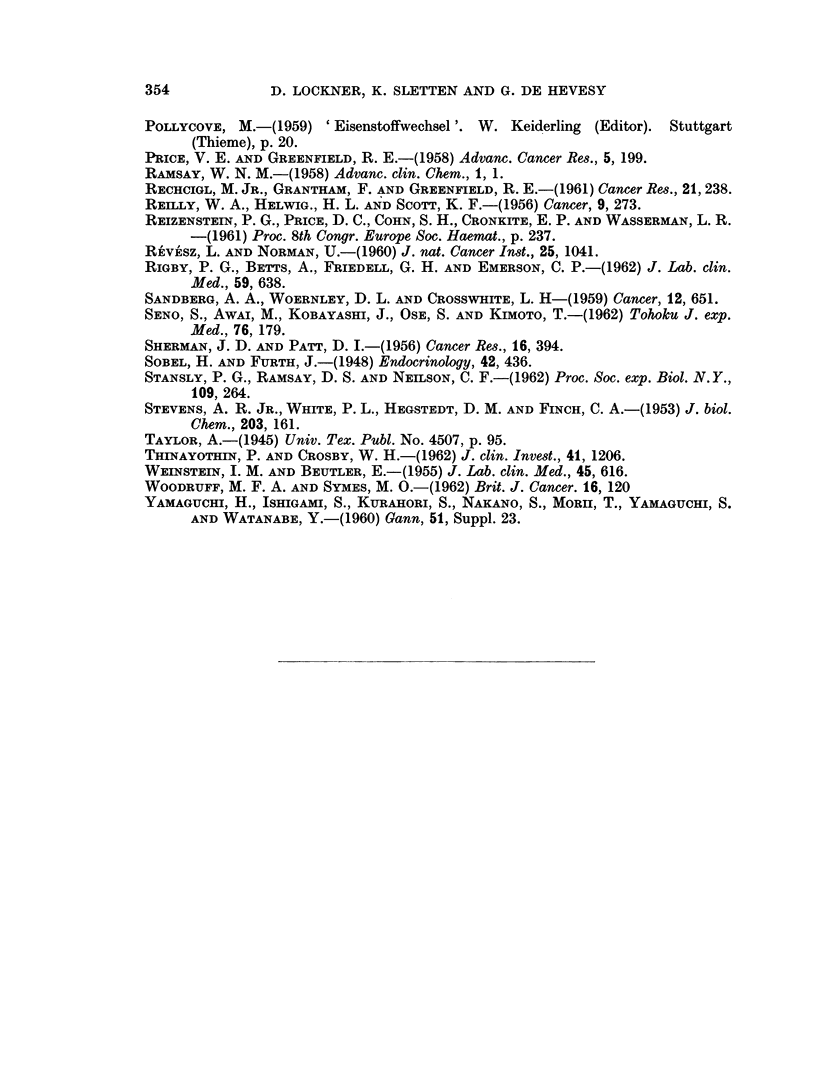# Studies on Cancer Anaemia

**DOI:** 10.1038/bjc.1963.47

**Published:** 1963-06

**Authors:** D. Lockner, K. Sletten, G. De Hevesy


### STUDIES ON CANCER ANAEMIA

### Organ Weights, Blood Values and Iron Metabolism in Normal and Tumour-bearing Mice

D. LOCKNER, K. SLETTEN AND G. DE HEVESY

*From the Institute of Organic Chemistry and Biochemistry, University of Stockholm, Sweden*

Received for publication March 4, 1963

In the course of our studies on cancer anaemia in humans (Lockner, 1960) it was felt that a clearer insight into the disturbances of body composition and iron metabolism accompanying cancer could be gained by studying experimental animals. We therefore studied mice bearing inoculated and spontaneous cancers. The anaemia in the animals studied was found to be caused by blood dilution ; increased iron storage in the reticulo-endothelial system could not be demonstrated, nor did the tumour play a prominent role in the elimination of erythrocytes from the circulation. A preliminary account of this work has been presented (Lockner, 1961 ; Hevesy and Lockner, 1962).

### METHODS

*Animals and tumours*

Adult C mice (Swiss albino mice from the Serum Institute in Copenhagen) and adult C3H mice (kindly supplied by Professor G. Klein, Institute for Tumour Biology, Stockholm) were used. The animals were fed with Salmonella controlled mouse pellets (Anticimex No. 210, Stockholm) and water *ad libitum.*

Some of the C mice were injected intramuscularly into one hind leg with about $2 \times 10^6$ freshly taken cells of an Ehrlich ascites tumour strain (ELD (Révész and Norman, 1960), kindly supplied by med. cand. U. Norman, Institute for Tumour Biology, Head : Prof. G. Klein), suspended in 0·1 ml. sterile saline. Between 10 and 20 days were allowed for tumour growth. The C3H mice used consisted partly of females bearing spontaneous mammary carcinomas, partly of young females without tumours and partly of healthy males. Animals with ulcerated tumours were discarded.

*Radioactivity*

$^{59}FeCl_3$ (5–20 $\mu c/\mu g$.Fe Oak Ridge National Laboratory) in 3·8 per cent ammonium citrate was used. Maximally 0·25 $\mu c$ $^{59}Fe$ in 0·1 ml. was injected intraperitoneally into groups of 5 to 10 animals for radioiron distribution studies. Other groups were quantitatively injected into a tail vein with 0·1 ml. mouse plasma containing radioiron previously incubated (30 minutes at 37° C.). An aliquot of each radioactive solution injected was set aside to be used as a standard.

At different time intervals after $^{59}Fe$ injection, groups of 5–10 animals were killed and the amount of radioactive iron determined in their organs. In this way the percentage of the administered as well as of the recovered radioiron was determined in blood, liver, spleen, kidneys, muscles, skin, skeleton, tumour and residual organs (all organs except those mentioned). To make it possible to distinguish between radioiron contained in the constituents of an organ and radioiron in the blood remaining in this organ at the death of the animal, the circulating blood of the mice was counterlabelled with $^{51}$Cr-labelled mouse erythrocytes of the same strain 15 minutes before killing the animals, this procedure giving at the same time the blood volume of the animals. The donor erythrocytes were washed with ACD-solution, incubated with $Na_2{}^{51}CrO_4$ (10–140 $\mu$c/$\mu$g. Cr) for one hour at room temperature, washed three times with ice cold saline and finally resuspended in their own plasma which had been saved. 0·1 ml. of this erythrocyte suspension containing 0·05–1·0 $\mu$c $^{51}$Cr was quantitatively injected into a tail vein, and an aliquot was taken as a standard. The animals were killed in Nembutal narcosis (3–5 mg. intraperitoneally) by exsanguination from the retro-ocular sinus (using heparin as anticoagulant). The organs were collected, immediately weighed, pooled and wet digested with sulphuric, nitric and perchloric acids. The skeleton was carefully freed from muscles and dry digested at controlled temperature in a muffle oven. The ash was several times extracted with 6 N HCl ; no significant iron loss occurred with this procedure. The iron content of the organ digests was determined with *o*-phenanthroline (Borei, 1943), their radioactivity was measured in a well-type scintillation counter (Ekco Ltd., England). The volume of all samples was identical. The radiations from $^{51}$Cr and $^{59}$Fe were distinguished by pulse discrimination techniques (Weinstein and Beutler, 1955). Counting was done sufficiently long to keep the counting error under $\pm$ 2 per cent. All measurements and determinations were done on duplicate samples.

For the plasma iron turnover studies, in which $^{59}$Fe bound to mouse plasma was intravenously injected, an effort was made to select animals having about the same body weight and tumour size. The animals were killed at different time intervals after injection, blood was taken as described, plasma was obtained by centrifugation, weighed and assayed for radioactivity. One animal served for one value. The blood volume was taken to be practically identical. Therefore radioactivity per gram plasma was plotted directly in correlation to time on semilogarithmic paper for calculation of plasma iron half-time.

To be able to study cohorts of cells furnished in a short time interval, reutilisation of radioiron from destroyed erythrocytes and recirculation of radioiron from organs was blocked by injection of inactive iron. Healthy and tumour-bearing C mice were first injected intraperitoneally with radioiron. Starting with the first day after radioiron injection inactive iron was given (Ferrigen kindly supplied by Astra, Södertälje) by the intraperitoneal route, 1 mg. daily per mouse. At suitable intervals groups of 5 to 10 animals were exsanguinated. Haemin was prepared from their blood by the acid-acetone method (London, Morell and Kassenaar, 1960), three times purified, wet digested and the specific iron activity determined as described.

In a long term experiment $^{59}$Fe was injected intraperitoneally into 10 C mice. After 97 days the animals were killed by exsanguination and the radioiron distribution determined. From part of the pooled blood, haemin was prepared as described, and from part of the pooled livers and muscles ferritin and haemosiderin were isolated (Gabrio, Shoden and Finch, 1953 ; Loftfield and Bonnichsen, 1956 ; von Ehrenstein and Lockner, 1959), wet digested and the specific activity of the iron contained in them determined.

*Blood values*

Haematocrit was determined as microhaematocrit by taking the blood directly from the retroocular sinus of the mice. Haemoglobin was measured as cyanmethaemoglobin in a Beckman DU spectrophotometer. Standardisation was done with Acuglobin (Ortho Pharm. U.S.A.). Plasma iron was determined according to Ramsay (1958). All blood values were determined in duplicate.

All experimental values are, if possible, given as the mean value $\pm$ one standard deviation of the mean. The significance of the difference between two mean values was calculated making use of variance analysis according to Bonnier and Tedin (1940).

### RESULTS AND DISCUSSION

*Organ weights*

The body and organ weights from C3H mice bearing spontaneous mammary carcinomas and from C mice bearing inoculated solid-growing Ehrlich ascites tumours and their respective controls are given in Table I and II. As the spontaneous mammary carcinomas of the C3H mice develop only in female animals and nearly 100 per cent of all females get tumours, it is difficult to find enough female controls of suitable weight and age. We succeeded in studying 7 such animals and their data are given together with those of 19 healthy males and 33 tumour-bearing females (Table I). The difference between the sum of the organ weights and the body weight given in the tables is due to blood taken from the animals when killing them.

Cancerous and control groups of C mice held the same mean weight when the tumour was injected and they still hold it when a 1 g. tumour has developed (Table II). This means that these animals feed up their tumours at the expense of their other body constituents, not by directing an eventual higher nutrient intake to develop the tumour as an extra organ (cf. Begg, 1958). The body weight of the cancerous C3H mice is higher than that of both control groups (Table I), which show no difference between each other. When however the tumour weight is subtracted from the body weight of the cancerous C3H mice all three groups hold equal weight. This fact makes a comparison between cancerous and control animals very suitable (Table I).

The liver and spleen weights of the cancer-bearing animals of both groups show a pronounced increase. The kidney weights of the cancer-bearing C mice were not changed, nor were those of the cancer bearing C3H mice compared to their female controls, while the male controls had a significantly higher kidney weight. The residual organs were unchanged in weight in cancer-bearing C mice, whereas they were lower in cancer-bearing C3H mice compared with both control groups. Skin weight was increased in cancer-bearing C mice and unchanged in C3H mice. Muscle weight was unchanged in both groups, only the male C3H mice showed a higher value. The weight of the skeleton was lower in both cancer groups (Table II).

The findings on liver and spleen weight confirm those of earlier investigators (cf. Begg, 1958; Andreini, Drasher and Mitchison, 1955; von Ehrenstein, 1958*a*; Rigby *et al.*, 1962; Woodruff and Symes, 1962). One of the main reasons for the increase in liver and spleen weight found in our animals seems to be a massive plasma cell or lymphocytic infiltration and reticulo-endothelial hyperplasia

TABLE I.—*Mean Values ± One Standard Deviation and Statistical Significance of the Difference Between Mean Values of Total Body and Organ Weights from 33 Female C3H Mice Bearing Spontaneous Mammary Carcinomas, 19 Healthy Male and 7 Healthy Female C3H Mice. "Total Body Corr." Means that the Tumour Weight has been Subttraced from the Body Weight of the Cancer-bearing Mice*

| | Male controls g. | Cancerous g. | Female controls g. | Male controls/ cancerous | Female controls/ cancerous | Male controls/ fem. controls |
|---|---|---|---|---|---|---|
| Total body | 24·9±1·9 | 28·5±4·3 | 23·6±2·7 | $p \eqsim 0\cdot001$ | $0\cdot01>p>0\cdot001$ | $p \eqsim 0\cdot2$ |
| Total body corr. | .. | .. | .. | $p \gg 0\cdot2$ | $p \gg 0\cdot2$ | .. |
| Liver | 1·642±0·262 | 1·924±0·396 | 1·546±0·252 | $0\cdot05>p>0\cdot01$ | $0\cdot05>p>0\cdot01$ | $p \gg 0\cdot2$ |
| Spleen | 0·120±0·037 | 0·319±0·140 | 0·157±0·041 | $0\cdot001 \gg p$ | $0\cdot2>p>0\cdot05$ | $0\cdot2>p>0\cdot05$ |
| Kidneys | 0·536±0·078 | 0·376±0·054 | 0·360±0·097 | $0\cdot001 \gg p$ | $p \gg 0\cdot2$ | $0\cdot001 \gg p$ |
| Residual organs | 5·185±0·755 | 4·794±0·631 | 5·620±0·980 | $0\cdot2>p>0\cdot05$ | $0\cdot01>p>0\cdot001$ | $0\cdot01>p>0\cdot001$ |
| Skin | 4·244±0·724 | 3·922±0·610 | 3·779±0·445 | $p \eqsim 0\cdot2$ | $p \gg 0\cdot2$ | $0\cdot2>p>0\cdot05$ |
| Muscles | 6·436±0·771 | 5·056±0·840 | 5·247±0·129 | $0\cdot001 \gg p$ | $p \gg 0\cdot2$ | $0\cdot01>p>0\cdot001$ |
| Skeleton | 5·769±1·138 | 4·635±0·418 | 6·554±0·120 | $0\cdot001 \gg p$ | $0\cdot001 \gg p$ | $0\cdot2>p>0\cdot05$ |
| Tumour | .. | 4·068±2·230 | .. | .. | .. | .. |

TABLE II.—*Mean Values ± One Standard Deviation of Total Body and Organ Weights from 50 Healthy Male C Mice and 26 Male C Mice bearing Solid-Growing Ehrlich Ascites Tumours. Statistical Significance of the Difference between the Mean Values is Given*

| | Controls g. | Cancerous g. | Significance of difference |
|---|---|---|---|
| Total body | 30·4±4·3 | 31·2±3·4 | $p \gg 0 \cdot 2$ |
| Liver | 1·658±0·264 | 1·974±0·234 | $0 \cdot 001 \gg p$ |
| Spleen | 0·155±0·075 | 0·291±0·073 | $0 \cdot 001 \gg p$ |
| Kidneys | 0·482±0·096 | 0·466±0·047 | $p > 0 \cdot 2$ |
| Residual organs | 6·994±1·600 | 6·792±0·939 | $p > 0 \cdot 2$ |
| Skin | 5·182±1·019 | 5·931±0·582 | $0 \cdot 001 > p$ |
| Muscles | 7·826±1·350 | 7·705±1·170 | $p > 0 \cdot 2$ |
| Skeleton | 6·570±1·128 | 6·062±1·330 | $0 \cdot 2 > p > 0 \cdot 05$ |
| Tumour | .. | 1·036±0·382 | .. |

in both organs, seen on histological examination.* Similar or identical results were obtained by other investigators (cf. Begg, 1958 ; von Ehrenstein, 1958*a* ; Baruah, 1958 ; Sandberg, Woernley and Crosswhite, 1959 ; Friedell, Sherman and Sommers, 1960 ; Rigby *et al.*, 1962). The eventual contribution of the increased amounts of erythropoietic tissue, which were seen by the microscope, to the weight increase in these organs is discussed later. The fact that the changes found in liver and spleen weight and their histological appearance are identical in tumour-bearing C3H and C mice suggests that they are not caused by the inoculation of foreign cancer cells, as could be the case for the C mice (cf. Old *et al.*, 1960).

The growth-impulse to the spleen seems to be transmitted by the blood, as it is possible to cause an increase in spleen weight by injecting blood from cancerous animals into healthy ones (Crossley *et al.*, 1955 ; von Ehrenstein, 1958*a* ; Stansly, Ramsay and Nielson, 1962). There seems, on the other hand, to exist a correlation between the amount of necrotic tissue in a tumour and the increase it causes in spleen weight (Sherman and Patt, 1956), while the effect on liver weight is usually slight or absent. The spleen weight was found to correlate significantly ($0 \cdot 01 > p > 0 \cdot 001$) with tumour weight, but not the liver weight. In human subjects who had died from cancer no general increase in liver or spleen weight could be found (Calo, 1932 ; Müller, 1932 ; Hevesy, unpublished observations), which is possibly explained by the cachexia usually accompanying heavy cancer growth. The same is true for animals that die spontaneously from cancer (cf. Begg, 1958 ; von Ehrenstein, 1958*a*).

The decrease of kidney weights in tumour-bearing C3H mice found by von Ehrenstein (1958*a*) seems to be a sexual difference, as we have found it too when we compared cancerous females with male controls, but not in comparison with female controls. Nor did C mice of identical sex show any difference.

The reduction in weight of the residual organs of the cancer-bearing C3H mice seems to be due to loss of fat (cf. Begg, 1958) from the attachments of the intestines, the guts contributing the biggest part in weight to the heading " residual organs ". The absence of any change in the C mice group may be due to the considerably smaller size of tumour and its shorter time of growth in these animals. We have no explanation for the increase in skin weight in the cancer-bearing C

* Thanks are due to Prof. G. Häggqvist, Stockholm, for interpreting the histological sections of the mouse organs.

TABLE III.—*Mean Values* ± *One Standard Deviation and Statistical Significance of the Difference Between Mean Values of Haematological Data From* 33 *Female C3H Mice Bearing Spontaneous Mammary Carcinomas*, 15 *Healthy Male and* 5 *Healthy Female C3H Mice*

| | Male controls | Cancerous | Female controls | Male controls/ cancerous | Female controls/ cancerous | Male controls/ fem. controls |
|---|---|---|---|---|---|---|
| Haemoglobin conc. g. % | 13·7±2·1 | 10·0±2·3 | 14·9±0·5 | $0{\cdot}001 \gg p$ | $0{\cdot}001 \gg p$ | $p > 0{\cdot}2$ |
| Haematocrit % | 44·7±6·1 | 32·9±6·0 | 49·4±1·2 | $0{\cdot}001 \gg p$ | $0{\cdot}001 \gg p$ | $0{\cdot}2 > p > 0{\cdot}05$ |
| Blood volume ml. | 2·1±0·4 | 2·8±0·9 | 1·8±0·2 | $0{\cdot}01 > p > 0{\cdot}001$ | $0{\cdot}05 > p > 0{\cdot}01$ | $0{\cdot}2 > p > 0{\cdot}05$ |
| Plasma volume ml. | 1·16±0·20 | 1·88±0·78 | 0·90±0·10 | $0{\cdot}001 > p$ | $0{\cdot}01 > p > 0{\cdot}001$ | $0{\cdot}05 > p > 0{\cdot}01$ |
| Erythrocyte volume ml. | 0·92±0·18 | 0·87±0·17 | 0·86±0·06 | $p > 0{\cdot}2$ | $p \gg 0{\cdot}2$ | $p \gg 0{\cdot}2$ |
| Total haemoglobin mg. | 280±56 | 256±34 | 261±14 | $0{\cdot}2 > p > 0{\cdot}05$ | $p \gg 0{\cdot}2$ | $p > 0{\cdot}2$ |
| MCHC % | 30·7±0·8 | 30·1±3·8 | 30·5±0·8 | $p \gg 0{\cdot}2$ | $p \gg 0{\cdot}2$ | $p \gg 0{\cdot}2$ |

mice. The higher muscle weight in the male C3H mice is probably a sex difference. The lower skeleton weight in both cancer groups may possibly be caused by osteoporosis, but no experiments were done to confirm this explanation.

*Haematological data*

The haematological data for both groups of mice are shown in Tables III and IV. It can be seen from the haemoglobin concentration and haematocrit values that the cancer-bearing animals of both groups are anaemic. The difference between cancerous and control values is highly significant. The blood volume is significantly and markedly ($\sim$ 30 per cent) increased in both groups of cancerous animals. This increase is due solely to an increase in the plasma volume, which is highly significant, whereas the erythrocyte volume is unchanged. As a consequence the cancerous animals of both groups have an unchanged total haemoglobin content. The mean corpuscular haemoglobin concentration (MCHC) is in both groups unchanged, which means that the anaemia is normochromic.

Price and Greenfield (1958) have pointed out that part of the reported changes of blood volume in cancerous individuals may be caused by loss of weight induced by the growth of cancer, and that correction for this loss tends to change these values to normal ones. The unchanged body weight of the cancerous C mice in spite of a 1 g. growth could at least in part be caused by fat loss. On the other hand it is questionable if the badly vascularized tumour tissue in both types of cancers studied has a higher blood content than fat. So, for example, the mean-sized tumour in our C3H mice comprises nearly 20 per cent of their body weight. We consequently conclude that weight changes are of minor importance in bringing about the blood volume changes in the animals studied by us. As, at the same time, a normal total haemoglobin and erythrocyte volume in combination with a heavy increased plasma volume are found, we must call this anaemia a dilution anaemia.

TABLE IV.—*Mean Values ± One Standard Deviation of Haematological Data from 35 Healthy Male C Mice and 35 male C Mice Bearing Solid Growing Ehrlich Ascites Tumours. Statistical Significance of the Difference Between the Mean Values is given*

| | Controls | Cancerous | Degree of significance |
|---|---|---|---|
| Haemoglobin conc. g. per cent | $13 \cdot 6 \pm 0 \cdot 9$ | $11 \cdot 4 \pm 2 \cdot 7$ | $0 \cdot 001 \gg p$ |
| Haematocrit per cent | $44 \cdot 1 \pm 2 \cdot 7$ | $36 \cdot 9 \pm 8 \cdot 2$ | $0 \cdot 001 \gg p$ |
| Blood volume ml. | $2 \cdot 1 \pm 0 \cdot 3$ | $2 \cdot 7 \pm 0 \cdot 6$ | $0 \cdot 001 \gg p$ |
| Plasma volume ml. | $1 \cdot 18 \pm 0 \cdot 19$ | $1 \cdot 69 \pm 0 \cdot 46$ | $0 \cdot 001 \gg p$ |
| Erythrocyte volume ml. | $0 \cdot 93 \pm 0 \cdot 13$ | $0 \cdot 96 \pm 0 \cdot 24$ | $p > 0 \cdot 2$ |
| Total haemoglobin mg. | $282 \pm 32$ | $303 \pm 23$ | $0 \cdot 2 > p > 0 \cdot 05$ |
| MCHC per cent | $30 \cdot 9 \pm 1 \cdot 7$ | $30 \cdot 8 \pm 2 \cdot 2$ | $p \gg 0 \cdot 2$ |

Taylor (1945) found that haemoglobin concentration decreased more rapidly than total haemoglobin fell, at the same time as the blood volume increased in his cancer-bearing mice ; but these animals did not show a constant total haemoglobin. The author used however only a semiquantitative method to determine the blood volume. Others (Sobel and Furth, 1948 ; Reilly, Helwig and Scott, 1956 ; van Ebbenhorst-Tengbergen and Mühlbock, 1958 ; von Ehrenstein, 1958*b*) found in mice and rats the same as we did. Similar results were obtained in patients with cancer of the uterine cervix (Lockner, 1960), while the values in other types of cancer differ (cf. Price and Greenfield, 1958).

Practically nothing is known about the underlying causes of the plasma volume increase in cancerous animals. In contrast to Sobel and Furth (1948) a decrease in plasma protein concentration in cancerous animals was found by us (Hevesy and Lockner, 1962), as by others in humans (Bodansky, 1956; Hammer, 1961). Rechcigl, Grantham and Greenfield (1961) demonstrated a higher intake of water and NaCl in tumour-bearing rats, which would partly explain the decreased plasma protein content. One is therefore inclined to seek the disturbance leading to plasma volume increase in the cancerous animals in mechanisms regulating sodium chloride excretion, such as an increase of aldosterone secretion or related mechanisms (cf. Moore, 1962).

FIG. 1.—Haemoglobin concentration and haematocrit values from cancer-bearing C mice at different time intervals after tumour inoculation. Each point represents the mean of 5 animals.

These results raise the question why the organism does not compensate for this blood dilution by an increased production of haemoglobin. Erslev (1955) showed in rabbits that artificial blood dilution did not increase erythropoiesis. The same seems to be the case in our cancerous mice. As will be discussed, the the life span of the erythrocytes in these animals is shortened and erythropoiesis increased. This increase leads to a complete compensation for the increased erythrocyte destruction, in that the total haemoglobin is kept normal; no compensation however is achieved for the blood dilution. Decreased haemoglobin concentration caused by an expanded plasma volume does not seem to be an adequate stimulus for erythropoiesis. Here the necessary oxygen supply to the tissues is obviously secured by haemodynamic adjustments.

Belcher and Simpson (1960) described sudden haemoglobin falls in rats bearing inoculated tumours when the tumour was only the size of a pea. As the underlying mechanism the authors discovered destruction of a large part of the circulating erythrocytes, possibly caused by a virus which could be transferred to other, non-tumour-bearing, animals. We were interested to see if this mechanism was in operation in our animals too. 160 C mice were injected with Ehrlich ascites tumour as described and at daily intervals, starting the day after tumour inoculation, groups of 5 animals were killed and haemoglobin concentrations as well as haematocrit values were determined. The blood volume was determined 14

and 26 days after tumour inoculation in 10 mice each by means of $^{51}Cr$ labelled mouse erythrocytes. Fig. 1 shows the haemoglobin concentration and haematocrit values. As can be seen, no sudden fall could be observed. The blood volume two weeks after the start of the experiment was 2·85 ± 0·20 ml. (SD) and at 26 days after cancer injection 3·10 ± 0·51 ml. (SD) ; at this time the tumours of the 10 mice in which the blood volume was determined had a mean weight of 9·73 g. After the 30th day of the experiment the tumours started to ulcerate and bleeding occurred ; this was accompanied by a heavy fall in haematocrit and haemoglobin concentration not indicated in the curve.

The continuous character of the decrease in haemoglobin concentration with growth of the tumour is further emphasised by the fact that haemoglobin concentration correlates with the tumour weight in all C mice studied with a significance of $0{\cdot}05 > p > 0{\cdot}01$ and for the C3H mice with $0{\cdot}001 > p$. Thus no mechanism as described by Belcher and Simpson (1960) seems to operate in our animals.

FIG. 2.—Percentage of $^{59}Fe$ recovered incorporated into circulating erythrocytes from cancer-bearing C3H and C mice and their respective controls at different time intervals after radioiron injecton. Each point represents the mean of 6 to 10 animals.

*Radioiron distribution*

In these and all the other experiments reported on radioiron distribution, mice which were always in about the same state of tumour development at varying intervals after radioiron injection, were studied.

*Blood*

Radioiron incorporation into the circulating erythrocytes of tumour-bearing C3H and C mice and their respective controls is shown in Fig. 2. Blood taken 6 hours after tracer application was corrected for plasma radioactivity. At the later points no plasma radioactivity could be measured. It can be seen that the tumour-bearing mice incorporate more radioiron into their erythrocytes than their respective controls. Furthermore, the curve of the cancerous C3H mice shows an early radioactivity peak at 3 days after radioiron injection. The cancerous C mice do not show this phenomenon. If however the reutilization of radioiron liberated from destroyed erythrocytes and the continuous release of radioiron from previously activated iron depots are blocked by the injection of inactive iron and, for greater accuracy, the activity of purified haemin is measured, then even the cancer-bearing C mice show this early peak in the blood radioactivity compared with their controls (Fig. 3).

The higher incorporation of radioiron into the blood of cancer-bearing as compared with control mice indicates that the cancer mice direct a bigger part of their plasma iron into the circulating erythrocytes. This means that cancer mice make more haemoglobin than control mice ; their erythropoiesis is increased in order to compensate, as will be shown later, for increased red blood cell destruction. Similar results were obtained by others (von Ehrenstein, 1958*b* ; Moore *et al.*, 1961 ; Otsuji, 1962).

FIG. 3.—Specific activity of purified blood haemin from tumour-bearing C mice and control C mice at different time intervals after radioiron injection. To block radioiron reutilization daily intraperitoneal injections of 1 mg. inactive iron were given.

Similar early peaks of blood radioactivity as reported here were found by Berlin, Lawrence and Lee (1951) in polycythaemic humans, by Berlin and Lotz (1951) in rats and by Neuberger and Niven (1951) in rabbits repairing bleeding anaemias, followed in later years by many more observations. von Ehrenstein (1958*b*) in studying the same mouse tumours as we did by means of [2-$^{14}$C] glycine came to identical conclusions. These findings must be interpreted as part of the cells furnished with the label decaying earlier than the bulk of the erythrocytes, thus representing a short-living erythrocyte population. Except that these populations were observed under conditions of stimulated erythropoiesis not much more is known about them. It is not even known with certainty if the normal human or animal produces naturally short-living erythrocytes, which we have no method to demonstrate unequivocally yet (London *et al.*, 1950 ; Evans, 1954 ; Ambs, 1960).

*Skeleton*

The bone constituents being relatively stable, the changes in non-circulating radioiron activity with time in the skeleton in these experiments are considered to reflect merely bone marrow radioactivity. As shown in Fig. 4 the bone marrow contains between 9 and 18 per cent noncirculating radioiron 6 hours after radioiron administration. Cancer bearing C3H as well as C mice exhibit much lower values than their respective controls. At subsequent observation points all values decline, the cancer curve always lying under the control curve. At the end of the observation period some increase of the skeleton radioactivity was again noted.

At 6 hours after radioiron injection between 10 and 35 per cent of the radioiron recovered is already incorporated into the circulating erythrocytes. Thus a big portion of the radioiron has already, after entering the bone marrow by way of the plasma, left it again incorporated into erythrocytes. A better picture of the dynamic state of the blood compartment at this moment is therefore given if we add blood and bone marrow figures at 6 hours after radioiron injection. At this time C3H controls contain about 24 per cent and C3H cancers 33 per cent recovered radioiron in these organs. Nearly 30 per cent more radioiron is thus

FIG. 4.—Percentage of $^{59}Fe$ recovered as noncirculating radioiron in the bone marrow from tumour-bearing C3H and C mice and their respective controls at different time intervals after radioiron injection. Each point represents the mean of 6 to 10 animals.

directed to blood production in the cancerous animal. A similar relationship exists in the case of C mice as can easily be seen from the blood and skeleton figures. These findings, as well as the heavier decay of both cancer skeleton curves as compared with their controls, are signs of the increased erythrocyte production in the cancerous animals.

The later increase in the bone marrow curves may have two explanations. One is the destruction of erythrocytes, with the radioiron liberated from their haemoglobin recirculating or directly laid down (von Ehrenstein and Lockner, 1959) in the bone marrow. The other is the slow redistribution of radioiron previously laid down in the depot organs. As the bone marrow of the cancerous animals is emptied more completely of iron by the increased erythropoiesis, in spite of a higher destruction rate of the erythrocytes in the cancerous animals, less radioiron remains there.

*Liver*

The change in the concentration of noncirculating radioiron in the liver of both groups of animals with time is shown in Fig. 5. The curve of the cancerous C3H mice lies considerably lower than that of their controls ; the cancerous C mice show some tendency to be lower still. The liver of the cancerous mice however has a mean weight increase (cf. Tables I and II) of about 15 per cent. Taking these facts in combination we can state that the total as well as the specific uptake of radioiron into the liver is considerably lower in cancer-bearing animals than in controls. This is in contrast to the reticulo-endothelial hyperplasia seen in this organ. Similar results were obtained by von Ehrenstein (1958*b*). In C3H mice a certain fall of the radioiron with time can be observed, which is possibly due to redistribution of iron. No such fall can be seen in the *C* mice. Both control curves show no fall in activity between 6 hours and 3 days, which could be indicative of erythropoiesis going on in the liver. Such a fall is however observed in the curves of the cancer-bearing animals, though the decrease is so small that it may only be caused by biological and experimental variations or by redistribution of iron.

FIG. 5.—Percentage of $^{59}Fe$ recovered as noncirculating radioiron in the liver of tumour-bearing C3H and C mice and their respective controls at different time intervals after radioiron injection. Each point represents the mean of 6 to 10 animals.

*Spleen*

As shown in Fig. 6, the spleen of the cancerous animals of both groups contains more noncirculating radioiron than the control spleen throughout the whole experiment. After a steep fall, between 6 hours and 3 days, of cancer and control spleen, the radioactivity remains at very low values, with a slight tendency to increase after 39 days.

From plasma iron turnover studies, which will be discussed later, we know that the primary distribution of radioiron is finished after a few hours. So that, if all erythrocytes were made in the bone marrow, the decrease in radioiron there should be followed by a corresponding increase in blood radioiron. Comparing the values between 6 hours and 3 days we find that at least twice as much iron is accumulated in the erythrocytes as is leaving the bone marrow in the same time interval. This discrepancy can be explained in two ways. One is that radioiron is redistributed from other organs and passes the bone marrow without our being able to see it. Organs releasing iron are shown to be the liver, skin, residual organs and muscles. Another possibility is that erythrocytes are furnished at extramedullary sites. This is histologically proven for the spleen of the mouse (Jacobson *et al.*, 1950). The early decay in radioiron activity in the control animals is good evidence for effective erythropoiesis going on in this organ. The 6 hour radioiron uptake of the cancerous spleen is higher by about 10 per cent than the corresponding control values ; the early decay runs parallel to them. This would mean that the cancerous spleen shows a somewhat increased erythropoiesis. On histological examination an increase in erythrocyte precursors in the cancerous spleen as compared with control spleen can in fact be seen. Considering however that the weight of the cancerous spleen is increased by more than 100 per cent its specific erythropoietic activity is thus calculated to be decreased by about 50 per cent. That means that this accessory erythropoietic tissue is not very effective in making erythrocytes. The weight increase already discussed is mainly due to this tissue and the other cell elements already described. This fact helps us to understand why the later horizontal part of the spleen curve runs considerably

FIG. 6.—Percentage of $^{59}$Fe recovered as noncirculating radioiron in the spleen of tumour-bearing C3H and C mice and their respective controls at different time intervals after radioiron injection. Each point represents the mean of 6 to 10 animals.

higher than the control curve. There may already be increased erythrocyte destruction a short time after radioiron injection, but because of the early horizontal part of the curve it is more convincing to interpret it as an increased iron storage in the enlarged spleen. The slight increase in radioactivity occurring at the end of the observation period may however be caused by accumulation of decaying erythrocytes.

### *Skin, residual organs, muscles and kidneys*

The changes in noncirculating radioiron with time in skin, residual organs, and muscles are shown in Fig. 7. It can be seen that each of these organs is nearly as effective in storing radioiron as the liver. Nearly all cancer curves run somewhat lower than the control curves, especially if we allow for weight differences, which indicates that more iron is used for erythropoiesis in the cancerous animals. The steep early fall as seen in most of the curves could indicate erythropoiesis, but as no erythropoietic function is usually associated with these organs we consider it indicates redistribution of depot iron. The late increase observed in some of the curves seems to be caused by destruction of labelled erythrocytes occurring in these organs or uptake of iron liberated from erythrocytes destroyed in other organs.

The kidneys never contain more than 2 per cent of the recovered radioiron and do not show any changes in their radioiron content.

*Tumours*

The content of noncirculating radioiron in spontaneous mammary carcinoma tissue from C3H mice at different intervals after radioiron injection is shown in Fig. 8. The upper curve shows the measured radioactivity in the different experiments carried out, the irregularity of the curve being due to variation in tumour size. The middle curve shows the tumour radioactivity calculated for the mean tumour weight of the C3H mice in all experiments, which is 4·07 g. The lower curve shows the specific activity of the noncirculating tumour iron.

FIG. 7.—Percentage of $^{59}Fe$ recovered as noncirculating radioiron in skin, residual organs and muscles of tumour-bearing C3H and C mice and their respective controls at different time intervals after radioiron injection. Each point represents the mean of 6 to 10 animals.

Price and Greenfield (1958) have found that the main part of the erythrocytes destroyed in the cancerous rats and mice studied by them is destroyed and laid down in the tumour.

It can be seen from the middle curve in Fig. 8 that 7 per cent of the recovered radioiron is accumulated as noncirculating radioiron in the tumour 6 hours after injection. As not many labelled erythrocytes can be destroyed in so short a time we consider this iron to have got there via the plasma. In the course of 39 days this accumulation increases to about 18 per cent. That means that about 11 per cent of the recovered radioiron accumulates in the tumour between 6 hours and 39 days after radioiron injection. There are two possible ways for the radioiron to get there : (*a*) by redistribution, via plasma, of iron taken up by other organs soon after iron injection, or iron liberated from labelled erythrocytes destroyed in other organs, (*b*) by direct destruction of labelled erythrocytes in the tumour tissue.

(*a*) 20–30 per cent of the recovered radioiron leaves the liver, residual organs, muscles and skin in the actual time interval by redistribution. As this exchange takes place via the plasma, and as the tumour takes up 7 per cent radioiron from the plasma, this redistribution increases tumour activity by 1–2 per cent. The

FIG. 8.—Percentage of $^{59}Fe$ recovered as noncirculating radioiron and specific activity of noncirculating tumour-iron in the spontaneous mammary carcinomas of C3H mice at different time intervals after radioiron injection. The upper curve gives the values of the individual experiments, the middle curve is a recalculation of the same values on the mean tumour weight of 4·07 g. Each point represents the mean of 6 to 10 animals.

life time of the erythrocytes in the cancerous C3H mice is, as will be shown, of the order of 14–20 days. That means that in the observation period discussed here (39 days) all erythrocytes are destroyed about twice. As about 70 per cent of the injected radioiron has been incorporated (via the plasma) into the circulating erythrocytes, this amount must be redistributed via the plasma again when these cells are destroyed. As we have discussed above, 7 per cent of the plasma iron is directly built into the tumour tissue. This means that even 7 per cent of this 70 per cent, that is about 5 per cent radioiron, is by redistribution built into tumour tissue from iron liberated when all erythrocytes are destroyed once. As this happens about twice within 39 days, the total amount built in is about 10 per cent. To these 10 per cent the 1–2 per cent discussed above have to be added. Thus 11–12 per cent of the recovered radioiron could theoretically reach the tumour via the plasma from redistribution of depot iron and radioiron liberated and redistributed from destroyed erythrocytes. That is just the amount actually laid down in the tumour in this time interval.

(*b*) Let us assume that all iron found in the tumour originates from destroyed erythrocytes laid down there. Greenfield, Sterling and Price (1960) found that about 1 per cent of the radioiron deposited in the tumour by means of labelled erythrocytes leaves it again per day. This loss would amount in the actual case where 11 per cent accumulated in 39 days to about 1 per cent. In total the tumour would thus contain 11 + 1 per cent, that is 12 per cent at 39 days. As discussed above, this 12 per cent accumulated during the life time of two cell populations containing about 70 per cent of the recovered radioiron. That means that when one erythrocyte population is broken down about 6 per cent iron is laid in the tumour, thus about 10 per cent of the erythrocytes destroyed are destroyed in the tumour if all points discussed under (*a*) were not right.

Concluding we cannot find that the mechanism proposed by Price and Greenfield (1958) is operating to a greater degree in the C3H mice studied by us. A similar result was obtained in a patient with a cancer of the uterine cervix (Hevesy and Lockner, 1962) and by others in rats (Belcher and Simpson, 1960) and hamsters (Rigby *et al.*, 1962).

There still remains however the question of where the erythrocytes are destroyed in cancerous mice. It can be shown (von Ehrenstein and Lockner, 1959 ; Hughes Jones, 1961) that erythrocyte destruction in normal rabbits occurs approximately proportionally to the distribution of the reticulo-endothelial

FIG. 9.—Percentage of $^{59}Fe$ recovered as noncirculating radioiron in 1 g. spontaneous mammary carcinoma of C3H mice and 1 g. solid growing Ehrlich-ascites tumour of C mice at different time intervals after radioiron injection. Each point represents the mean of 6 to 10 animals.

system in the body, the bone marrow playing a very prominent role in this process. Owing to the reutilization and redistribution processes going on in the type of experiments described here it is very difficult to draw any definite conclusion. The curves shown suggest, however, that erythrocyte destruction seems to take place in bone marrow, spleen, skin and muscles under normal conditions and that in the cancerous state these organs show about the same behaviour with only minor quantitative differences, with perhaps additional destruction in the liver too.

The slower increase of the specific activity of the tumour iron as compared with the middle curve in Fig. 8 shows that, with time, iron of lower specific activity is laid down in the tumour.

The accumulation of noncirculating radioiron in the solid growing Ehrlich tumours of our C mice is shown in Fig. 9, compared with that in spontaneous mammary carcinomas of C3H mice and calculated per 1 g. tumour weight. It can be seen that the two curves are practically identical and run horizontally up to 15 days. At 39 days after iron injection the C3H curve increases. We were not able to study Ehrlich tumours for such a long time, because ulceration and bleeding occurred from the tumours and the animals died. It is of interest that the activity of both tumours studied in accumulating radioiron is so similar. This suggests that even in C mice the mechanism which shortens the life time of the erythrocytes in cancerous mice is not mainly cell removal by the tumour.

*Total body distribution*

The radioiron distribution between the main body components of control and cancerous C3H and C mice is seen in Fig. 10 and Fig. 11. In both cancer groups the mean tumour weight was taken. It can be seen from Fig. 10 that the carcass of the tumour-bearing C3H mice contains only about half as much non-circulating radioiron as the carcass of the control C3H mice. The greater part of this missing radioiron is found in the blood, a lesser part in the tumour. With time, some radioiron is shifted over from the blood to tumour and carcass. The tumour-bearing C mice show a similar picture (Fig. 11) the difference being however not so pronounced as for the C3H mice. Up to 15 days the tumour-bearing C mice show no shift of radioiron from blood to carcass or tumour.

FIG. 10.—Percentage $^{59}Fe$ recovered as circulating radioiron in erythrocytes and as non-circulating radioiron in carcass and tumour of cancer-bearing and control C3H mice at different time intervals after radioiron injection. Carcass means total body radioiron minus blood and tumour-radioiron.

These findings indicate that the main change in the iron metabolism of the cancerous animal is a shift from depot (carcass) iron to the blood, as a result of increased erythropoiesis, and partly to the tumour. This result is in contrast to suggestions made by Heilmeyer and Keiderling (1959) that, in a way similar to that postulated by them for the anaemia of infection, an activated reticulo-endothelial system competes for the plasma iron, using it for detoxicating purposes, thus not leaving enough over for blood production. We have histological evidence for an activated reticulo-endothelial system, as already discussed, but it does not seem to be activated with respect to iron-avidity. Analogous results have been reported by others (Otsuji, 1962 ; Seno *et al.*, 1962).

One could be inclined to interpret the later part of the cancerous C3H mice curve, where some iron shifts from blood to tumour and carcass, in the sense of Heilmeyer and Keiderling (1959). But this is not correct, because here we are no longer studying the initial plasma iron distribution. This false impression is caused by the accelerated turnover of the cancerous erythrocytes in contrast to the slow turnover of tissue iron. That this effect is not observed in cancer-bearing C mice is due to the shorter observation period corresponding with the longer erythrocyte lifetime in these mice (*vide infra*).

FIG. 11.—Percentage of $^{59}$Fe recovered as circulating radioiron in erythrocytes and as non-circulating radioiron in carcass and tumour of cancer-bearing and control C mice at different time intervals after radioiron injection. Carcass means total body radioiron minus blood and tumour-radioiron.

*Influence of type of radioiron application*

In the radioiron distribution studies discussed so far, for quantitative and technical reasons the tracer was injected intraperitoneally. It was assumed that distribution would not differ markedly from administration by the intravenous route. In order to study eventual differences in distribution connected with the intravenous route, 6 control C mice were quantitatively injected intravenously into a tail vein with radioiron bound to mouse plasma and the radioiron distribution determined after 3 days. The results obtained are compared with a 3-day experiment on control C mice injected by the intraperitoneal route. The results are given in Table V without standard deviations because the organs were pooled for technical reasons.

TABLE V.—*Percentage of $^{59}$Fe Recovered in Circulating Haemoglobin and as Non-circulating Iron in Different Organs of Healthy C Mice* 3 *Days After Injecting Radioiron into* 10 *Mice Intraperitoneally and into* 6 *Mice Bound to Plasma Intravenously*

| | Intraperitoneally per cent | Intravenously per cent |
|---|---|---|
| Blood | 54·5 | 62·1 |
| Skeleton | 5·0 | 3·9 |
| Liver | 12·2 | 9·5 |
| Spleen | 0·0 | 0·0 |
| Kidneys | 0·2 | 0·7 |
| Residual organs | 17·0 | 6·1 |
| Skin | 4·9 | 11·3 |
| Muscles | 6·1 | 6·3 |

When the intraperitoneal route is used, the abdominal organs (liver and residual organs) contain about 14 per cent more of the recovered radioactivity than the corresponding organs after intravenous administration. This 14 per cent goes, when the intravenous route is used, in equal parts to the blood and skin instead.

Thinayothin and Crosby (1962) found that iron-dextran entered the small intestine via the serosa, being carried by phagocytes. Whether the transferrin-bound iron used by us is transported by the same mechanism is not known to us. The fact that we found an increase in liver uptake too, which the authors cited did not, suggest some difference in mechanism. The higher uptake in the residual organs explains the steep fall of their activity already discussed as redistribution but lessens their importance as depot organs. These findings have no implications on the results presented on the influence of cancer on iron metabolism, as comparisons were made between equally treated groups.

*Difference in radioiron distribution between male and female C3H mice*

As already discussed, it is difficult to get enough female control C3H mice for extended studies. Male C3H mice of about the same age and weight as the cancerous female mice were therefore used as controls in the radioiron distribution studies reported. In order to see how far this could lead to wrong conclusions, the radioiron distribution was compared 3 days after intraperitoneal iron administration between 4 female and 5 male control C3H mice. The results of this study are given in Table VI.

It can be seen that the radioiron distribution is practically identical. The female C3H mice contain less total body iron, even if the weight difference is allowed for, a fact the importance of which will be stressed below.

TABLE VI.—*Percentage of $^{59}Fe$ Recovered in Circulating Haemoglobin and as Noncirculating Radioiron in Different Organs of 4 Healthy Female and 5 Healthy Male C3H Mice 3 Days After Intraperitoneal Application of Radioiron. Total Body Iron Content, Haemoglobin Concentration, Haematocrit and Body Weight are given too.*

| | Females | Males |
|---|---|---|
| Percentage of $^{59}Fe$ recovered in | | |
| Blood | 53·5% | 56·9% |
| Skeleton | 2·0% | 1·0% |
| Liver | 14·5% | 13·3% |
| Spleen | 0·6% | 0·4% |
| Kidneys | 0·3% | 0% |
| Residual organs | 16·9% | 16·8% |
| Skin | 6·5% | 5·0% |
| Muscles | 5·9% | 6·6% |
| Total body iron | 1245 μg. | 1380 μg. |
| Haemoglobin concentration | 15·1 g.% | 15·5 g.% |
| Haematocrit | 49% | 49% |
| Body weight | 22 g. | 23 g. |

*Iron content and iron loss of healthy and cancer-bearing mice*

Tables VII and VIII show the noncirculating iron content of the organs of tumour-bearing C3H and C mice and their respective controls as well as their blood iron and total body iron.

There is no significant difference in total body iron between cancer-bearing and control C mice. The spleen of the cancer-bearing mice contains however significantly more iron and the residual organs significantly less than in the control animals. All other organs studied show no difference, nor does the body weight of the two groups show any statistical difference. No measurable noncirculating iron could be found in kidneys and skeleton.

TABLE VII.—*Mean Values ± One Standard Deviation of the Noncirculating Iron Content of the Organs, Blood Iron and Total Body Iron of* 10 *C Mice Bearing Solid Growing Ehrlich Ascites Tumours and* 35 *Control* C *Mice. No Standard Deviations for the Cancerous Mice are given as they consisted of One Group in which the Organs were Pooled. The Statistical Significance of the Difference between Mean Values is given too.*

| | Controls | Cancerous | Significance of difference |
|---|---|---|---|
| Blood | 959 ± 109 μg. | 1013 μg. | $0{\cdot}2 > p > 0{\cdot}05$ |
| Liver | 67 ± 12 μg. | 58 μg. | $0{\cdot}2 > p > 0{\cdot}05$ |
| Spleen | 6 ± 5 μg. | 24 μg. | $0{\cdot}001 \gg p$ |
| Residual organs | 220 ± 56 μg. | 169 μg. | $0{\cdot}01 > p > 0{\cdot}001$ |
| Skin | 174 ± 79 μg. | 142 μg. | $0{\cdot}2 > p > 0{\cdot}05$ |
| Muscles | 149 ± 18 μg. | 155 μg. | $p > 0{\cdot}2$ |
| Kidneys | None measurable | None measurable | .. |
| Skeleton | None measurable | None measurable | .. |
| Tumour | .. | 34 μg. | .. |
| Total body iron | 1575 ± 124 μg. | 1595 μg. | $p \gg 0{\cdot}2$ |
| Body weight | 30·9 ± 5·7 g. | 33·6 ± 4·5 g. | $0{\cdot}2 > p > 0{\cdot}05$ |
| Tumour weight | .. | 1·327 ± 0·276 g. | .. |

The increased iron content of the spleen is related to the increased spleen weight already discussed. Plasma cell infiltration, increase of reticulo-endothelial tissue, effective and ineffective erythropoiesis as well as a possible increase of erythrocyte destruction in this organ can be named as reasons for this effect. As the liver weight is increased for similar reasons except for erythropoiesis, and as this organ contains a little less iron in the cancerous animals, we are inclined to seek the reason for the increase in spleen iron mainly in the ineffective erythropoiesis and partly in the increased erythrocyte destruction going on in this organ under the influence of cancer. The lower iron content of the residual organs and the skin of the cancerous animals is in contrast to an increase in cancerous C3H mice. No explanation can be given for this observation.

The absence of measurable iron in the skeleton suggests that no chemically measurable iron pool exists in the bone marrow.

In C3H mice we find similar results as in C mice (Table VIII). Some changes are more pronounced, possibly caused by the greater activity of the tumour in bringing about metabolic changes typical of the cancerous growth, as it is nearly four times bigger than in cancerous C mice. Furthermore the development of the tumour is much slower in C3H than in C mice. Most important here, as in the earlier comparisons, are the control-female values compared with the cancerous, as the latter are of female sex too. Even here we can see that in spite of an increased liver weight no more iron can be found in this organ. Spleen iron is markedly increased, for reasons already discussed. The residual organs in spite of decreased weight, and skin, muscles, and kidneys in spite of unchanged weight show significant increases in their iron content. As a consequence we find a heavily increased (32 per cent) total body iron in the cancerous group. Even if

TABLE VIII.—*Mean Values* ± *One Standard Deviation of the Non-circulating Iron Content of the Organs, Blood Iron and Total Body Iron of* 33 *Female C3H Mice Bearing Spontaneous Mammary Carcinomas,* 17 *Healthy Male and* 4 *Healthy Female C3H Mice. No Standard Deviations for some data of the Female Controls are given as these organs were pooled. The Statistical Significance of the Difference between Mean Values is given too. "Total Body Corr." Means that the Tumour Iron has been Subtracted from the Total Body Iron of the Cancerous Mice*

| | Male controls | Cancerous | Female controls | Male controls/cancerous | Female controls/cancerous | Male controls/fem. controls |
|---|---|---|---|---|---|---|
| Blood | 952±178 μg. | 871±117 μg. | 864±37 μg. | $0\cdot2>p>0\cdot05$ | $p\gg0\cdot2$ | $p>0\cdot2$ |
| Liver | 70±27 μg. | 95±33 μg. | 71 μg. | $0\cdot01>p>0\cdot001$ | $0\cdot2>p>0\cdot05$ | $p\gg0\cdot2$ |
| Spleen | 5±2 μg. | 40±18 μg. | 12 μg. | $0\cdot001\gg p$ | $0\cdot001\gg p$ | $0\cdot01>p>0\cdot001$ |
| Residual organs | 154±65 μg. | 229±29 μg. | 121 μg. | $0\cdot001\gg p$ | $0\cdot05>p>0\cdot01$ | $p>0\cdot2$ |
| Skin | 97±51 μg. | 205±22 μg. | 89 μg. | $0\cdot001\gg p$ | $0\cdot001\gg p$ | $p\gg0\cdot2$ |
| Muscles | 99±27 μg. | 146±54 μg. | 88 μg. | $0\cdot01>p>0\cdot001$ | $0\cdot05>p>0\cdot01$ | $p\gg0\cdot2$ |
| Kidneys | 5±5 μg. | 19±11 μg. | None measurable | $0\cdot001>p$ | $0\cdot01>p>0\cdot001$ | $0\cdot2>p>0\cdot05$ |
| Skeleton | None measurable | None measurable | None measurable | .. | .. | .. |
| Tumour | .. | 253±112 μg. | .. | .. | .. | .. |
| Total body iron | 1380±206 μg. | 1857±235 μg. | 1245±21 μg. | $0\cdot001\gg p$ | $0\cdot001>p$ | $p>0\cdot2$ |
| Total body corr. | .. | .. | .. | $0\cdot001>p$ | $0\cdot001>p$ | .. |
| Body weight | 24·6±1·8 g. | 28·5±4·3 g. | 22·3±0·6 g. | $0\cdot01>p>0\cdot001$ | $0\cdot01>p>0\cdot001$ | $0\cdot05>p>0\cdot01$ |
| Tumour weight | .. | 4·068±2·230 g. | .. | .. | .. | .. |

we subtract the tumour iron from the total body iron, thus eliminating the difference in weight between the groups (total body corr. in Table VIII), the higher iron content of the cancerous animals is still highly significant (22 per cent) Neither cancerous nor control C3H mice contain measurable quantities of depot iron in their bone marrow. It is interesting that residual organs, skin and muscles contain far more iron than the liver in both cancerous and control mice. When we combine the data of iron and organ weight given in this paper we are able to calculate the specific iron content of the different organs. For the C mice we find that liver and spleen have the highest iron concentration, but only that of the spleen increases in cancer. The C3H mice data show that here also the spleen contains the highest iron concentration, a great increase taking place in cancer. The liver remains relatively unchanged, though marked increases are noted for residual organs, skin, muscles and kidneys. The tumour of the C3H mice has the highest iron concentration next to the spleen, but this is only half as much as in the last mentioned organ. An increase in liver-iron and a decrease in spleen-iron in cancer was found by others (Yamaguchi *et al.*, 1960).

With regard to the heavily increased iron content of the cancerous C3H mice, we were interested to know if this increase was achieved by decreased excretion or increased absorption of iron or a combination of both. By giving radioiron to an organism and measuring its total radioiron content at different time intervals, some indication can be obtained of the iron loss occurring in it. Studies were made with this technique in mice (Stevens *et al.*, 1953 ; von Ehrenstein and Hevesy, 1959) and in man (Finch, 1959). Recently the total body counter came into use for this purpose (cf. Reizenstein *et al.*, 1961). If the loss of radioiron is to be an indicator of the amount of total iron lost by the organism, the iron given must mix completely with the body iron. That this is presumably not the case could be shown by Finch (1959) for the human and is seen from Table IX for the mouse, 97 days after administration of radioiron.

TABLE IX.—*Specific Activities of some Tissue Protein Fractions* 97 *Days After Intraperitoneal Radioiron Injection into Control C Mice. Preparations obtained from Pooled Organs of* 10 *Animals*

| | |
|---|---|
| Blood haemin . . | 13·7 counts/min./μg.Fe |
| Liver ferritin . . . | 13·9 ,, ,, ,, |
| Liver haemosiderin . | 11·4 ,, ,, ,, |
| Muscle ferritin . . | 18·8 ,, ,, ,, |
| Muscle haemosiderin . | 13·2 ,, ,, ,, |

The specific activities of liver haemosiderin, muscle ferritin, and muscle haemosiderin are considered to deviate significantly from the other values given. Thus, when the iron is given by injection, radioiron loss from mice merely indicates loss of labile body iron. By comparing the total amount of radioiron recovered after different time intervals, in our studies on distribution of radioiron, with the amount of radioiron injected, we find the percentage loss of labile body iron in the groups of animals investigated. The curves are given for cancerous C3H and C mice and their respective controls in Fig. 12. The curves show irregularities, as the values given were always obtained by summing up all the separately determined organ activities, which necessarily aggravates eventual mistakes. It can be seen however that the cancerous C3H mice lose considerably more labile radioiron than all the other groups. This is in contrast to the greatly

TABLE X.—*Plasma Iron Turnover in Cancer Bearing C3H and C Mice and their respective Controls. Values in Brackets Indicate Number of Animals used for the Particular Value. All Other Values were taken from Tables or figures given in this paper. C3H Controls consisted of Males*

| | Plasma Fe μg. % | $T\frac{1}{2}$* min. | Ht† % | Total Hb mg | Pl. vol. ml | In-corp.‡ % | Plasma iron turnover: mg. Fe/hr/1 RBC | Plasma iron turnover: μg. Fe/day | Plasma iron turnover: μg. Fe inc./day | mg. Hb synthesized/day | RBC§ life-time (days) |
|---|---|---|---|---|---|---|---|---|---|---|---|
| Control C mice | 239(15) | 68(23) | 44·1 | 282 | 1·18 | 56 | 2·22 | 41·5 | 23·2 | 6·8 | 41·5 |
| Cancerous C mice | 203(18) | 67(33) | 36·9 | 303 | 1·69 | 65 | 2·52 | 50·8 | 33·0 | 9·7 | 31·2 |
| Control C3H mice | 282(4) | 95(10) | 44·7 | 280 | 1·16 | 56 | 1·83 | 34·5 | 19·3 | 5·7 | 49.2 |
| Cancerous C3H mice | 172(5) | 52(11) | 32·9 | 256 | 1·88 | 72 | 3·25 | 62·0 | 44·7 | 13·1 | 19·5 |

* Plasma iron half-time.

† For turnover calculations the haematocrit values were corrected for venous/body haematocrit ratio by the factor 0·92 (Brown, Hopper and Wennesland, 1957) and for 2 per cent plasma trapping (Chaplin and Mollison, 1951).

‡ Percentage maximal radioiron incorporation into the circulating erythrocytes.

§ Total Hb/Hb synthesized per day.

increased body iron content of the cancerous C3H mice and indicates that these mice must absorb considerably more iron than healthy animals.

*Plasma iron turnover*

Plasma iron turnover was studied in cancer-bearing C3H and C mice and their respective controls (Table X). The disappearance of trace amounts of $^{59}Fe$ from the plasma of cancer-bearing and control C mice showed no difference. A shorter half-time was however found for the cancerous C3H mice as compared with their controls. The plasma iron turnover was calculated according to a modification of Huff's method (Huff, 1960). Plasma iron concentration was determined in other animals than those used for the turnover studies. It can be seen from Table X that the plasma iron concentration decreases in cancer,

FIG. 12.—Total body radioiron activity recovered at different time intervals after radioiron injection into control and cancer-bearing C3H and C mice. Each point represents the mean of 6 to 10 animals.

as has been found by others (cf. Price and Greenfield, 1958). The C mice with their smaller cancers show a smaller effect. In spite of an unchanged half-time and a decreased plasma iron the cancerous C mice are able to increase their iron turnover. This they do by increasing iron incorporation into their erythrocytes. In the cancerous C3H mice this mechanism is working along with a shortened half-time, but is even more pronounced. This is a compensating mechanism which the human cannot use to this degree, a fact which is discussed in greater detail by Hevesy and Lockner (1962). The iron turnover calculated per hour per litre of erythrocytes is, for normal mice, about three times that for humans (Lockner, 1960), a fact which corresponds well with the three times longer life span of erythrocytes in humans. By knowing the amount of iron transported in the plasma per day, and its percentage incorporated into the erythrocytes, we are able to deduce the amount of haemoglobin synthesized per day (Table X). Dividing the amount of total haemoglobin present in the circulation by the amount synthesized per day, the life time of the erythrocytes can be calculated. The values found for control C and C3H mice correspond quite well with those found by von Ehrenstein (1958*c*), who, using [2-$^{14}$C]glycine found values between 40 and 46 days. Even the values for the cancerous mice show good correlation. von Ehrenstein found for TE mice bearing solid growing Ehrlich cancer 26 days; these tumours were however about twice as big as ours. For cancer-bearing C3H mice a value of 14 days was found by this author.

It is not possible to calculate from human plasma iron turnover values the lifetime of human erythrocytes (Pollycove, 1959), because a usually unknown amount of iron returns from the bone marrow. It is therefore surprising that our values, obtained by the iron method, correspond so well to direct determinations. This allows us to conclude that in contrast to the human, no measurable amounts of iron return from the bone marrow into the plasma, an assumption which is supported by the fact that we cannot find measurable quantities of noncirculating iron in the bone marrow, suggesting that no measurable "labile bone-marrow pool" exists in the mouse. Even a considerably smaller amount would have been detected.

### SUMMARY

Organ weights, blood values, $^{59}$Fe distribution and turnover, as well as iron content were studied in C mice bearing inoculated solid-growing Ehrlich ascites tumours (ELD) and in C3H mice carrying spontaneous mammary carcinomas and in their respective controls.

Liver and spleen weights were found to be significantly increased, but skeleton weights were decreased in cancerous mice of both groups.

The cancerous mice of both groups showed a normochromic anaemia caused by blood dilution, as plasma- and blood-volumes were increased, whilst erythrocyte volume and total haemoglobin remained unchanged. The anaemia develops continuously in relation to tumour weight. As the erythrocyte life span is shortened in these animals, this organism is able to compensate for this shortening, but not for the blood dilution. Both groups of mice produce an extra population of short-living erythrocytes under the influence of cancer.

The cancerous mice show a shift of radioiron from carcass to blood in spite of an activated reticulo-endothelial system. Muscles, skin and residual organs are shown to be important iron depot organs besides the liver. It is shown that the tumours studied are scarcely active in destroying circulating erythrocytes within their tissues. The isotope studies confirm the erythropoietic function of the spleen, but in spite of greatly increased erythropoiesis in cancer this function of the spleen is not much increased.

The iron content of cancerous C3H mice is greatly increased in spite of an increased iron loss observed in these animals, which indicates considerably increased iron absorption. The increased iron content is laid down in the spleen, residual organs, skin and muscles.

Plasma iron turnover is increased in cancerous mice. The turnover figures allow the calculation of erythrocyte life times, giving 41·5 days for control C mice and 31·2 days for cancerous C mice. The corresponding values for C3H mice were 49·2 and 19·5 days. These values correspond quite well with those found by other methods. This finding allows the conclusion that no substantial amount of iron returns from the bone marrow after being laid down there via the plasma.

The study was made possible through the financial support given by the Swedish Cancer Foundation, the Jane Coffin Childs Memorial Fund for Medical Research and the Knut and Alice Wallenberg Foundation.

### REFERENCES

AMBS, E.—(1960) *Schweiz. med. Wschr.*, **90**, 413.

ANDREINI, P., DRASHER, M. L. AND MITCHISON, N. A—(1955) *J. exp. Med.*, **102**, 199.

BARUAH, B. D.—(1958) *Nature, Lond.*, **182**, 1455.

BEGG, R. W.—(1958) *Advanc. Cancer Res.*, **5**, 1.

BELCHER, E. H. AND SIMPSON, S. M.—(1960) *Brit. J. Cancer*, **14**, 224.

BERLIN, N. I., LAWRENCE, J. H. AND LEE, H. C.—(1951) *Science*, **114**, 385.

*Idem* AND LOTZ, C.—(1951) *Proc. Soc. exp. Biol. N.Y.*, **78**, 788.

BODANSKY, O.—(1956) *Med. Clin. N. Amer.*, **40**, 611.

BONNIER, J. AND TEDIN, O.—(1940) 'Biologisk Variationsanalys' Stockholm (Bonnier).

BOREI, H.—(1943) *Biochem. Z.*, **314**, 359.

BROWN, E. HOPPER, J. AND WENNESLAND, R.—(1957) *Ann. Rev. Physiol.*, **19**, 231.

CALO, A.—(1932) *Z. Krebsforsch.*, **37**, 151.

CHAPLIN, H. JR., AND MOLLISON, P. O.—(1951) *Blood*, **7**, 1227.

CROSSLEY, M. L., BRUCK, M., JENCKS, F. AND ALLISON, J. P—(1955) *Proc. Amer. Ass. Cancer Res.*, **2**, 11.

VAN EBBENHORST-TENGBERGEN, W. J. P. R. AND MÜHLBOCK, D.—(1958) *Brit. J. Cancer*, **12**, 81.

VON EHRENSTEIN, G.—(1958*a*) *Experientia*, **14**, 299.—(1958*b*) *Ark. Min.*, **13**, 199.—(1958*c*) *Acta physiol. scand.*, **44**, 80

*Idem* AND HEVESY, G.—(1959) *Acta haemat.*, **22**, 311.

*Idem* AND LOCKNER, D.—(1959) *Ibid.*, **22**, 129.

ERSLEV, A. J.—(1955) *Blood*, **10**, 616.

EVANS, R. L.—(1954) *Nature, Lond.*, **173**, 129.

FINCH, C. A.—(1959) *J. clin. Invest.*, **38**, 392.

FRIEDELL, G. H., SHERMAN, J. D. AND SOMMERS, S. C.—(1960) *Arch. Path.*, **70**, 363.

GABRIO, B. W., SHODEN, A. AND FINCH, C. A.—(1953) *J. biol. Chem.*, **204**, 815.

GREENFIELD, R. E., STERLING, W. R. AND PRICE, V. E.—(1960) *J. nat. Cancer Inst.*, **24**, 87.

HAMMER, O.—(1961) *Arzneimittelforschung*, **15**, 519.

HEILMEYER, L. AND KEIDERLING, W.—(1959) *Dtsch. med. Wschr.*, **84**, 724.

HEVESY, G. AND LOCKNER, D.—(1962) *Ark. Min.*, **19**, 303.

HUFF, R. L.—(1960) *Meth. med. Res.*, **8**, 35.

HUGHES JONES, N. L.—(1961) *Clin. Sci.*, **20**, 315.

JACOBSON, L. O., SIMMONS, E. L., BETHARD, W. F., MARKS, E. K. AND ROBSON, M. J.—(1950) *Proc. Soc. exp. Biol. N.Y.*, **73**, 455.

LOCKNER, D.—(1960) *Acta haemat.*, **24**, 186.—(1961) *Proc. 8th Congr. Europe Soc. Haemat.*, p. 294.

LOFTFIELD, R. B. AND BONNICHSEN, R. (1956) *Acta chem. scand.*, **10**, 1547.

LONDON, I. M., MORELL, H. AND KASSENAAR, A.—(1960) *Meth. med. Res.*, **8**, 136.

*Idem*, WEST, R., SHEMIN, D. AND RITTENBERG, D.—(1950) *J. biol. Chem.*, **184**, 351.

MOORE, F. D.—(1962) *Nord. med.*, **67**, 209.

MOORE, R. D., RUPP, J., MUHAW, V. AND SCHOENBERG, M. D.—(1961) *Arch. Path.*, **72**, 65.

MÜLLER, I.—(1932) *Zbl. allg. Path. path. Anat.*, **55**, 180.

NEUBERGER, A. AND NIVEN, J. S. F.—(1951) *J. Physiol.*, **112**, 292.

OLD, L. J., CLARKE, D. A., BENACERRAF, O. AND GOLDSMITH, M.—(1960) *Ann. N.Y. Acad. Sci.*, **88**, 264.

OTSUJI, S.—(1962) *Proc. Amer. Ass. Cancer Res.*, **3**, 350.

POLLYCOVE, M.—(1959) 'Eisenstoffwechsel'. W. Keiderling (Editor). Stuttgart (Thieme), p. 20.
PRICE, V. E. AND GREENFIELD, R. E.—(1958) *Advanc. Cancer Res.*, **5**, 199.
RAMSAY, W. N. M.—(1958) *Advanc. clin. Chem.*, **1**, 1.
RECHCIGL, M. JR., GRANTHAM, F. AND GREENFIELD, R. E.—(1961) *Cancer Res.*, **21**, 238.
REILLY, W. A., HELWIG., H. L. AND SCOTT, K. F.—(1956) *Cancer*, **9**, 273.
REIZENSTEIN, P. G., PRICE, D. C., COHN, S. H., CRONKITE, E. P. AND WASSERMAN, L. R. —(1961) *Proc. 8th Congr. Europe Soc. Haemat.*, p. 237.
RÉVÉSZ, L. AND NORMAN, U.—(1960) *J. nat. Cancer Inst.*, **25**, 1041.
RIGBY, P. G., BETTS, A., FRIEDELL, G. H. AND EMERSON, C. P.—(1962) *J. Lab. clin. Med.*, **59**, 638.
SANDBERG, A. A., WOERNLEY, D. L. AND CROSSWHITE, L. H—(1959) *Cancer*, **12**, 651.
SENO, S., AWAI, M., KOBAYASHI, J., OSE, S. AND KIMOTO, T.—(1962) *Tohoku J. exp. Med.*, **76**, 179.
SHERMAN, J. D. AND PATT, D. I.—(1956) *Cancer Res.*, **16**, 394.
SOBEL, H. AND FURTH, J.—(1948) *Endocrinology*, **42**, 436.
STANSLY, P. G., RAMSAY, D. S. AND NEILSON, C. F.—(1962) *Proc. Soc. exp. Biol. N.Y.*, **109**, 264.
STEVENS, A. R. JR., WHITE, P. L., HEGSTEDT, D. M. AND FINCH, C. A.—(1953) *J. biol. Chem.*, **203**, 161.
TAYLOR, A.—(1945) *Univ. Tex. Publ.* No. 4507, p. 95.
THINAYOTHIN, P. AND CROSBY, W. H.—(1962) *J. clin. Invest.*, **41**, 1206.
WEINSTEIN, I. M. AND BEUTLER, E.—(1955) *J. Lab. clin. Med.*, **45**, 616.
WOODRUFF, M. F. A. AND SYMES, M. O.—(1962) *Brit. J. Cancer.* **16**, 120
YAMAGUCHI, H., ISHIGAMI, S., KURAHORI, S., NAKANO, S., MORII, T., YAMAGUCHI, S. AND WATANABE, Y.—(1960) *Gann*, **51**, Suppl. 23.